# Genome-wide identification of *BAM* (β-amylase) gene family in jujube (*Ziziphus jujuba* Mill.) and expression in response to abiotic stress

**DOI:** 10.1186/s12864-022-08630-5

**Published:** 2022-06-13

**Authors:** Yaping Ma, Yaru Han, Xuerui Feng, Handong Gao, Bing Cao, Lihua Song

**Affiliations:** 1 School of Agriculture, Ningxia University, Yinchuan, 750021 China; 2 College of Forestry, Nanjing Forestry University, Nanjing, 210037 China; 3 Southern Tree Seed Inspection Center, Nanjing, 210037 China

**Keywords:** Jujube, Carbohydrate metabolism, Abiotic stress response, Evolutionary conservation, Starch-maltose interconversion, ZjBAM, Protein interaction

## Abstract

**Background:**

Elevated temperature and drought stress have substantial impacts on fruit quality, especially in terms of sugar metabolism and content. β-Amylase (*BAM*) plays a critical role in regulating jujube fruit sugar levels and abiotic stress response. Nevertheless, little is known about the regulatory functions of the *BAM* genes in jujube fruit.

**Results:**

Nine jujube *BAM* genes were identified, clustered into four groups, and characterized to elucidate their structure, function, and distribution. Multiple sequence alignment and gene structure analysis showed that all *ZjBAM* genes contain Glu-186 and Glu-380 residues and are highly conserved. Phylogenetic and synteny analysis further indicated that the *ZjBAM* gene family is evolutionarily conserved and formed collinear pairs with the *BAM* genes of peach, apple, poplar, *Arabidopsis thaliana*, and cucumber. A single tandem gene pair was found within the *ZjBAM* gene family and is indicative of putative gene duplication events. We also explored the physicochemical properties, conserved motifs, and chromosomal and subcellular localization of *ZjBAM* genes as well as the interaction networks and 3D structures of *ZjBAM* proteins. A promoter *cis*-acting element analysis suggested that *ZjBAM* promoters comprise elements related to growth, development, phytohormones, and stress response. Furthermore, a metabolic pathways annotation analysis showed that *ZjBAMs* are significantly upregulated in the starch and sucrose metabolism, thereby controlling starch-maltose interconversion and hydrolyzing starch to maltose. Transcriptome and qRT-PCR analyses revealed that *ZjBAMs* respond positively to elevated temperature and drought stress. Specifically, *ZjBAM1*, *ZjBAM2*, *ZjBAM5*, and *ZjBAM6* are significantly upregulated in response to severe drought. Bimolecular fluorescence complementation analysis demonstrated ZjBAM1-ZjAMY3, ZjBAM8-ZjDPE1, and ZjBAM7-ZjDPE1 protein interactions that were mainly present in the plasma membrane and nucleus.

**Conclusion:**

The jujube *BAM* gene family exhibits high evolutionary conservation. The various expression patterns of *ZjBAM* gene family members indicate that they play key roles in jujube growth, development, and abiotic stress response. Additionally, ZjBAMs interact with α-amylase and glucanotransferase. Collectively, the present study provides novel insights into the structure, evolution, and functions of the jujube *BAM* gene family, thus laying a foundation for further exploration of *ZjBAM* functional mechanisms in response to elevated temperature and drought stress, while opening up avenues for the development of economic forests in arid areas.

**Supplementary Information:**

The online version contains supplementary material available at 10.1186/s12864-022-08630-5.

## Background

Chinese jujube (*Ziziphus jujuba* Mill.) is a small deciduous tree in the family Rhamnaceae that originated in China, where it has been domesticated for > 7000 years. It is considered one of the oldest cultivated fruit trees in the world and is a commercially important species [1–3]. In China, jujube has a cultivation area of ~ 2 million hectares and an annual production of over 8 million tons, accounting for the primary source of income for millions of farmers [3, 4]. In addition, as a commercial crop, jujube has been introduced to more than 48 countries, including Korea, Iran, the United States, Australia, among other countries [4–6]. Jujube is highly nutritious and has been widely used as a food product and in traditional Chinese medicine (TCM) for many years. The fruit contains a variety of active components, including phenolics, flavones, polysaccharides, vitamin C, triterpenoid acid, saponins, α-tocopherol, and β-carotene [7, 8]. As such, it has demonstrated effectiveness in blood nourishment as well as in the treatment of different types of illnesses, including spleen diseases, diarrhea, skin infection, fever, and insomnia [7, 8]. Meanwhile, as a TCM it has exhibited anti-inflammatory, anti-cancer, gastrointestinal protective, antioxidant, immunomodulatory, and hypoglycemic properties [7, 9]. Moreover, owing to its outstanding endurance and adaptability to drought, as well as barren and salty soil, jujube is becoming increasingly prevalent in arid and semiarid lands, where it is considered a superfruit due to these unique advantages [4].

However, global climate change has markedly altered environmental conditions for plant growth and functioning [10]. Elevated temperatures and drought stress have significantly impacted the jujube fruit quality. Under severe drought (40% of the field water holding capacity) and elevated temperature interactive treatment, the jujube fruit weight decreases by 29%, whereas the organic acid content significantly increases and the soluble content decreases [11]. Moreover, elevated temperature (2.5 °C above normal temperature) significantly increases the total sugar content, sugar-organic acid ratio, anthocyanins, flavonoids, and carotenoids contents. Meanwhile, under drought stress, total sugar content and anthocyanin, flavonoid, and carotenoid contents are significantly reduced, whereas the chlorophyll and organic acid contents are increased [12, 13].

Sugar plays a vital role in the abiotic stress response of the jujube. The key genes regulating the jujube fruit sugar content are closely associated with sugar, organic acid, and secondary metabolism pathways. One such gene is *BAM* (β-amylase) [14]. BAMs (β-amylases; EC 3.2.1.2) play central roles in starch degradation and gene regulation by converting starch to maltose in multiple physiological processes including growth, development, and defense [15–17]. Indeed, *BAM* genes are ubiquitous in bryophyte, seedless vascular plant, gymnosperm, and angiosperm genomes [18]. The evolution of functional analysis revealed that the novel *BAM10* clade is absent in Arabidopsis. Meanwhile, BAM4 controls starch metabolism and is differentially regulated among various species [19]. *BAM* genes also play pivotal roles in abiotic stress tolerance by degrading starch and regulating soluble sugar accumulation in response to cold stress [20–22]. Considering that *BAM* gene expression and BAM protein activity are elevated in pear, blueberry, orange, tea tree, potato, and poplar under cold stress [23–28], this gene family may play key regulatory roles in jujube response to abiotic stress.

Although we have observed that elevated temperature and drought stress have significant effects on jujube fruit quality, the underlying response mechanisms remain unclear, particularly regarding the role of stress resistance genes. Accordingly, in the present study, we identified and characterized nine *ZjBAM* genes in the jujube genome and evaluated the network regulation of sugar metabolism, as well as transcriptome and expression patterns under elevated temperature and drought stress to establish the jujube fruit response to abiotic stress. Furthermore, the ZjBAMs protein-protein interaction network was analyzed and further validated by bimolecular fluorescence complementation (BiFC). The results of the present study provide novel insights, which would be helpful for future investigations, into the mechanisms by which jujube *BAM* gene family members regulate sugar metabolism in response to temperature, drought, and other abiotic stresses.

## Results

### Identification and characterization of *ZjBAM* genes in jujube

The HMMER and Pfam numbers (PF01373.19) were used to search BAM protein sequences in the jujube genome database and identify jujube *BAM* genes. The Expect (e) cutoff was set to 0.0001 to remove redundant sequences. Nine ZjBAM protein sequences were identified. The conserved Glyco_hydro_14 domain was confirmed with SMART and NCBI Batch CD-Search tools. The final gene sequences identified were named *ZjBAM1–ZjBAM9* (Table 1) based on the E-value order in the result of the HMMER profile. *ZjBAM9* was localized to chr 8, *ZjBAM7* was localized to chr 10, *ZjBAM2*, *ZjBAM3*, and *ZjBAM4* were localized to chr 11, and *ZjBAM*1, *ZjBAM5,* and *ZjBAM8* were localized to chr 12. The position of *ZjBAM6* could not be determined (Table 1).

**Table 1:** Characteristics of nine ZjBAM proteins

Gene name	Gene ID	CDS (bp)	Protein (aa)	Domains and position	Position	Physicochemical properties						Secondary structure				Subcellular location
						Formula	Molecular weight (kDa)	Theoretical pI	Instability index	Aliphatic index	Grand average of hydrophilicity (GRAVY)	Alpha helix (%)	random coil (%)	extended strand (%)	Beta turn (%)	
*ZiBAM1*	Zj.jz015515046	1644	547	Glyco_hydro_14 (88–505)	chr12: 8513131–8,516,292(+)	C_2705_H_4187_N_755_O_808_S_27_	61.16	7.96	34.02	68.63	−0.508	34.19	46.98	13.35	5.48	microbody
*ZiBAM2*	Zj.jz044849113	1752	583	Glyco_hydro_14 (122–545)	chr11:11966453–11,968,718(+)	C_2889_H_4443_N_805_O_854_S_36_	65.32	6.30	40.77	68.78	−0.388	39.28	43.05	11.84	5.83	cytoplasm
*ZiBAM3*	Zj.jz029235020	1650	549	Glyco_hydro_14 (117–537)	chr11:18000224–18,005,171(−)	C_2767_H_4180_N_768_O_811_S_24_	61.95	5.61	39.12	75.85	−0.342	31.15	48.09	15.66	5.10	microbody
*ZiBAM4*	Zj.jz029235021	2112	703	Glyco_hydro_14 (271–691)	chr11:17992552–17,998,025(−)	C_3482_H_5370_N_998_O_1051_S_37_	79.22	5.82	43.92	72.80	−0.484	36.13	46.23	12.23	5.41	nucleus
*ZiBAM5*	Zj.jz040945107	1683	560	Glyco_hydro_14 (95–519)	chr12:19124621–19,127,773(+)	C_2850_H_4333_N_787_O_797_S_38_	63.59	8.77	46.71	67.93	−0.280	30.89	50.36	12.86	5.89	microbody
*ZiBAM6*	Zj.jz220022001	1683	560	Glyco_hydro_14 (95–519)	unchr:60100583–60,103,735(+)	C_2850_H_4333_N_787_O_797_S_38_	63.59	8.77	46.71	67.93	−0.280	30.89	50.36	12.86	5.89	microbody
*ZiBAM7*	Zj.jz013313009	1683	560	Glyco_hydro_14 (65–486)	chr10:5521812–5,524,770(−)	C_2851_H_4367_N_743_O_848_S_21_	63.29	5.12	39.74	82.38	−0.400	38.04	43.75	12.68	5.54	cytoplasm
*ZiBAM8*	Zj.jz040841049	2091	696	Glyco_hydro_14 (263–667)	chr12:7290744–7,303,095(+)	C_3454_H_5273_N_965_O_1054_S_27_	78.25	5.53	41.31	72.76	−0.444	35.92	44.40	12.64	7.04	nucleus
*ZiBAM9*	Zj.jz004069034	1602	533	Glyco_hydro_14 (85–495)	chr08:16302112–16,304,795(+)	C_2610_H_4050_N_734_O_798_S_22_	59.2	7.27	44.98	71.73	−0.465	33.58	46.15	15.38	4.88	mitochondrial matrix

The physicochemical properties of the nine ZjBAM proteins were analyzed. The CDS lengths were in the range of 1602–2112 bp, and the protein lengths were in the range of 533–703 aa. The molecular formulas of the predicted ZjBAM proteins indicated that their elemental composition primarily included C, H, N, O, and S. Their molecular weights and isoelectric points were in the ranges of 59.2–79.22 kDa and 5.12–8.77, respectively. Their aliphatic indices were in the range of 68.63–82.3; hence, most were thermostable. According to the instability calculations and GRAVY, all proteins except ZjBAM1, ZjBAM3, and ZjBAM7 were unstable (> 40), and all were hydrophilic (< 0). ZjBAM1, ZjBAM3, ZjBAM5, and ZjBAM6 were localized to microbodies, ZjBAM2 and ZjBAM7 were localized to the cytoplasm, ZjBAM9 was localized to the mitochondria, and ZjBAM4 and ZjBAM8 were localized to the nuclei (Table 1).

### Conserved domain alignment, motif, and structural analyses of *ZjBAM* gene family

**Fig. 1:**
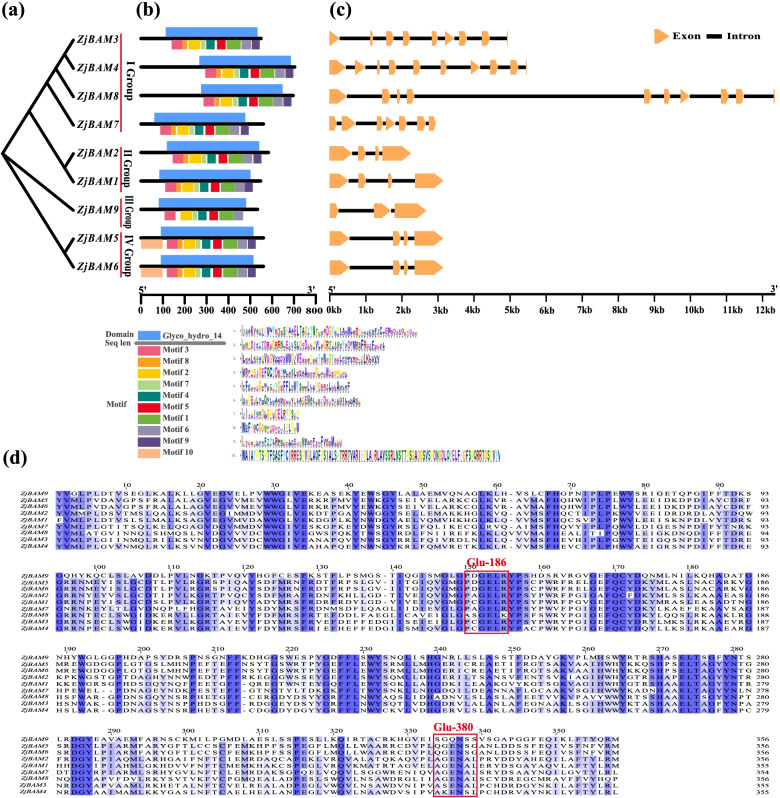
Bioinformatics analyzes of *ZjBAM* genes family. **a** Phylogenetic evolution of *ZjBAM* genes family. **b** Conserved domain and motif analysis of *ZjBAM* genes family. **c** Gene structure analysis of *ZjBAM* genes family. **d** Multiple sequence alignment of ZjBAM family amino acid, blue shading indicates highly conservative substitutions. Red box represents the two catalytic residues Glu-186 and Glu-380

The phylogenetic tree, conserved domains and motifs, and exon-intron structures of the *ZjBAM* gene family were combinatorically constructed. The nine different *ZjBAM* gene sequences were used to plot a phylogenetic tree. *ZjBAM3*, *ZjBAM4*, *ZjBAM8*, and *ZjBAM7* were clustered into group I, *ZjBAM1* and *ZjBAM2* were clustered into group II, *ZjBAM9* was in group III, and *ZjBAM5* and *ZjBAM6* were clustered into group IV (Fig. 1a). Each gene contained a Glyco_hydro_14 conserved domain at the positions shown in Fig. 1b. Ten conserved motifs and 20 amino acid residues were identified using MEME. All genes contained motifs 1–9 and were localized to a conserved domain region (Fig. 1b).

To characterize and elucidate the structural diversity of the *ZjBAM* genes, we analyzed the gene exon-intron structures using the Gene Structure Display Server program (Fig. 1c). The structural analysis revealed that the coding regions of all *ZjBAM* genes were interrupted by 2–9 introns, and all members of each group had similar structures. Group I had the most introns (nine in *ZjBAM4* and *ZjBAM8*, eight in *ZjBAM3*, and six in *ZjBAM7*) followed by groups II and IV. *ZjBAM1*, *ZjBAM2*, *ZjBAM5*, and *ZjBAM6* each contained three introns, while *ZjBAM9* in group III had only two (Fig. 1c). An analysis of the intron-exon structure showed that the *ZjBAM* gene family was evolutionarily conserved. Multiple sequence alignments of the ZjBAM amino acids disclosed that the Glyco_hydro_14 domain and the catalytic residues Glu-186 and Glu-380 were highly conserved across all gene family members (Fig. 1d).

### Phylogenetic analysis of *ZjBAM* genes

**Fig. 2:**
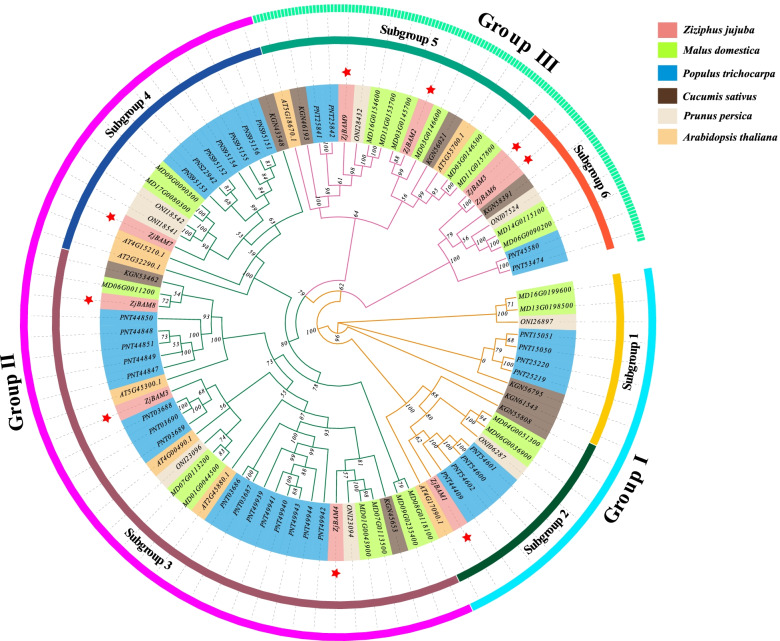
Phylogenetic tree of 90 *BAM* genes from *Ziziphus jujuba*, apple (*Malus domestica*), poplar (*Populus trichocarpa*), cucumber (*Cucumis sativus*), peach (*Prunus persica*), and *Arabidopsis thaliana*, created with MEGAX using the neighbor-joining method

Ninety *BAM* genes from *Z.jujuba*, apple (*Malus domestica*), poplar (*Populus trichocarpa*), cucumber (*Cucumis sativus*), peach (*Prunus persica*), and *Arabidopsis thaliana* were used to construct a phylogenetic tree (Fig. 2). These genes were classified into groups I, II, and III, and each of these was further divided into two subgroups. *ZjBAM1* was assigned to subgroup 2, *ZjBAM3*, *ZjBAM4*, and *ZjBAM8* to subgroup 3, *ZjBAM7* to subgroup 4, *ZjBAM2* and *ZjBAM9* to subgroup 5, and *ZjBAM5* and *ZjBAM6* to subgroup 6 (Fig. 2). The *BAM* genes in jujube, apple, poplar, cucumber, peach, and *A. thaliana* were expanded into the foregoing groups and subgroups. This indicates that these genes may have similar functions in the growth and development.

### Chromosomal localization, tandem duplication, and *cis*-acting element analyses of the *ZjBAM* gene family

**Fig. 3:**
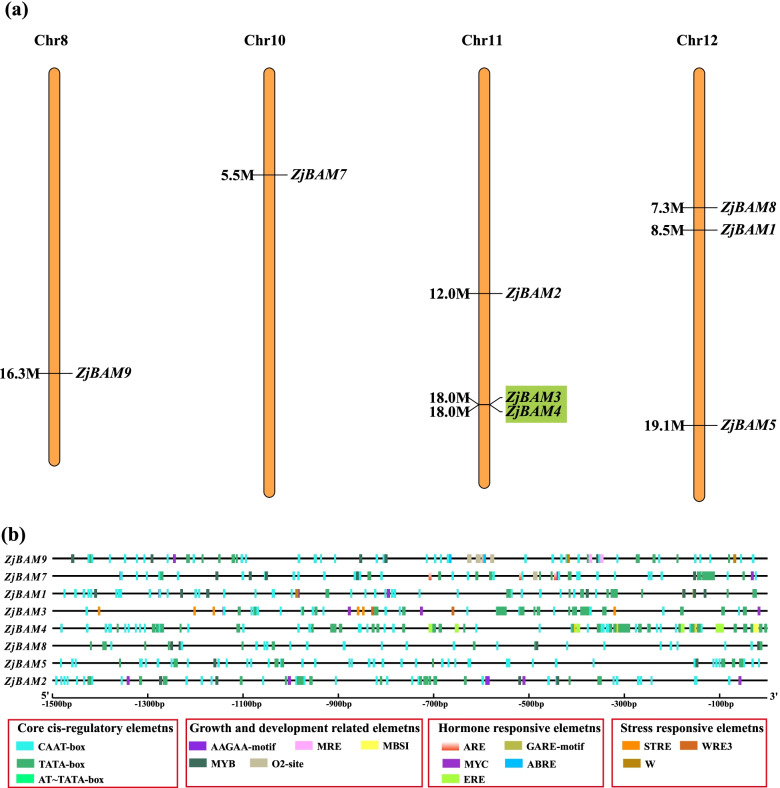
Chromosomal distribution and Cis-acting elements analysis of *ZjBAM* family. **a** Chromosomal localization of eight *ZjBAM* gene family members, the green box represents a pair of tandem genes *ZjBAM3* and *ZjBAM4*. **b** Promoter cis-acting element of *ZjBAM* genes

The chromosomal position of the identified *ZjBAM* genes was determined with the Mapchart software. Eight *ZjBAM* genes were located on four chromosomes in *Z. jujuba* (Fig. 3a). These included *ZjBAM9* on chr 8, *ZjBAM7* on chr 10, *ZjBAM2*, *ZjBAM3*, and *ZjBAM4* on chr 11, and *ZjBAM8*, *ZjBAM1*, and *ZjBAM5* on chr 12. A tandem gene pair (*ZjBAM3* and *ZjBAM4*) was detected with the MCScanX tool and was recognized as a marker on chr 11 (Fig. 3a, green highlight). The Ka/Ks ratio between *ZjBAM3* and *ZjBAM4* was < 1.0; this indicates that the gene pair probably underwent strong purifying selection for retention.

A 1500 bp upstream of each *ZjBAM* gene family was selected as a promoter region and searched with PlantCARE. The predicted *cis*-elements were classified mainly as core *cis*-regulatory-, growth and development-related-, phytohormone-responsive-, and stress-responsive elements (Fig. 3b). Each *ZjBAM* member contained a typical core promoter element TATA-box and an enhancer element CAAT-box, which enhances gene expression. The AAGAA-motif was found in *ZjBAM1* and *ZjBAM2*. The light-responsive element MRE was identified in the *ZjBAM9* promoter. MBSI is a flavonoid biosynthetic gene-regulating element occurring in the *ZjBAM4* promoters. O2-site is a zein metabolism-regulating element in the *ZjBAM9* and *ZjBAM7* promoters. The DNA synthesis ribonucleotide reductase enzyme element MYB and the phytohormone-responsive element MYC were observed in the *ZjBAM1*, *ZjBAM*2, *ZjBAM*5, *ZjBAM*7, *ZjBAM8,* and *ZjBAM9* promoters. The phytohormone-responsive elements ARE, GARE-motif, ABRE, ERE were identified in the *ZjBAM7*, *ZjBAM1*, *ZjBAM9*, *ZjBAM4* promoters, respectively. STRE, W, and WRE3 are environmental stress-responsive elements and were found in the *ZjBAM3* and *ZjBAM1* promoters (Fig. 3b).

### Intragenomic and intergenomic collinearity analysis

**Fig. 4:**
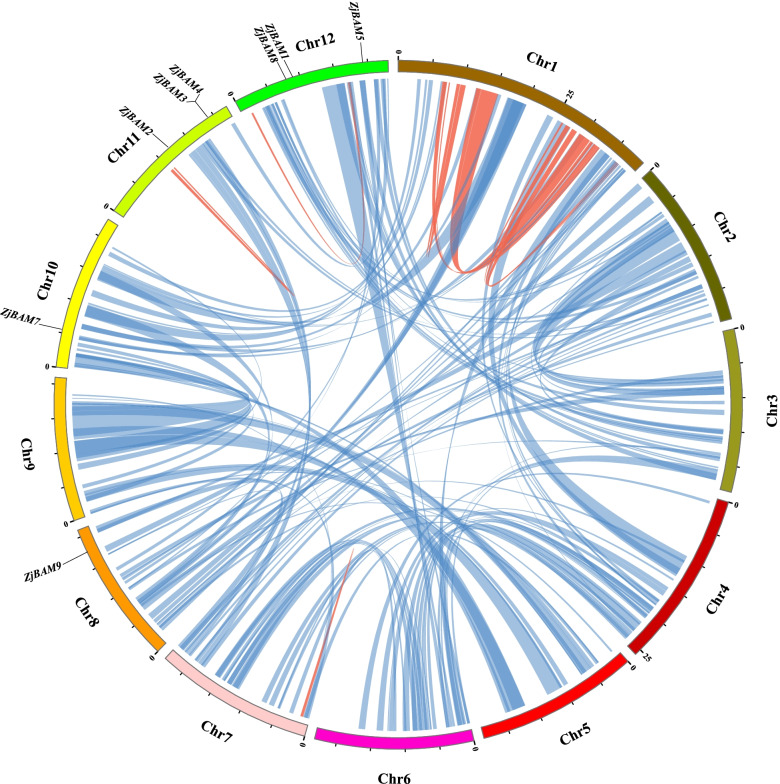
Intergenomic synteny relationship between the *ZjBAM* genes in the jujube genome. Red and blue lines indicate the collinear gene pairs within intrachromosomal and interchromosomal, respectively

**Fig. 5:**
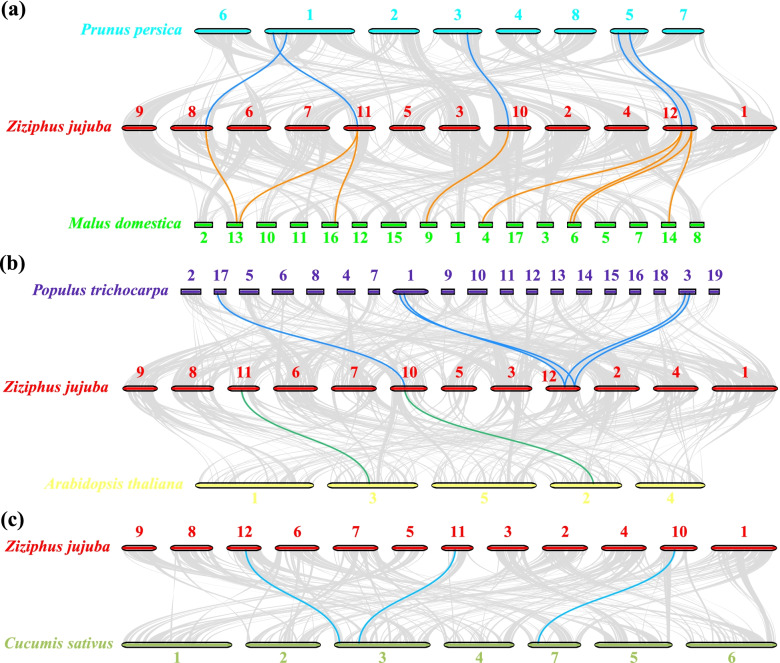
Synteny analysis of the *BAM* genes among jujube, apple (*Malus domestica*), poplar (*Populus trichocarpa*), cucumber (*Cucumis sativus*), peach (*Prunus persica*), and *Arabidopsis thaliana*. **a** Collinear *BAM* family gene pairs between peach and jujube and between jujube and apple. **b** Collinear *BAM* family gene pairs between poplar and jujube and between jujube and *A. thaliana*. **c** Collinear *BAM* family gene pairs between jujube and cucumber. Colored lines highlight the colinear gene pair

Intergenomic synteny analysis revealed different linear relationships among jujube chromosomes. A total of 1645 collinear gene pairs were detected in the intrachromosomal and interchromosomal regions; however, there was no collinearity among *ZjBAM* family members (Fig. 4). Intergenomic collinearity was analyzed to investigate genetic divergence and gene duplications of the *BAM* genes among jujube and peach, apple, poplar, *A. thaliana*, and cucumber. Five *ZjBAM* gene family members were collinear with five peach *BAM* genes and eight apple *BAM* genes (Fig. 5a; Table S1). In addition, three *ZjBAM* gene family members were collinear with five poplar *BAM* genes, and two were collinear with two *A. thaliana BAM* genes (Fig. 5b; Table S1). Similarly, three *ZjBAM* gene family members were collinear with three cucumber *BAM* genes (Fig. 5c; Table S1). A comparative genomic analysis showed that the *BAM* family genes were more collinear between jujube and apple, peach, and poplar than between jujube and *A. thaliana* and cucumber. Hence, duplicated genes might have been altered or lost during the evolution of the different species.

### Sugar metabolism regulation by *ZjBAM* gene family members and expression profiles in the transcriptome

**Fig. 6:**
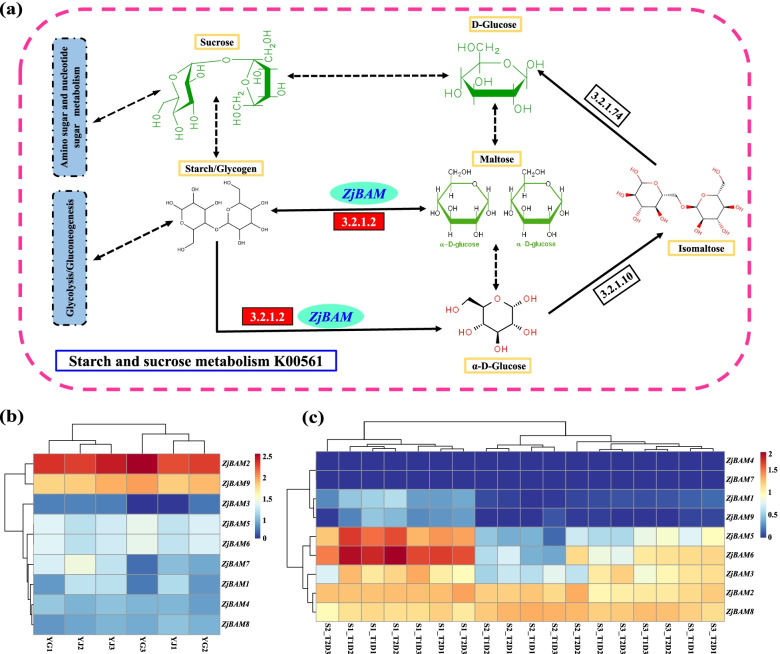
*ZjBAM* genes regulation and expression. **a** BAM in the regulating of the starch and sucrose metabolism pathways. Red box representative gene significantly upregulated. **b** Expression profiles of *ZjBAM* family genes in the grafting (YJ) and root tiller (YG) propagation of ‘Lingwuchangzao’ jujube fruit transcriptome. **c** Expression profiles of *ZjBAM* family genes under elevated temperature and drought transcriptome

Transcriptome analysis of the various propagation modes of ‘Lingwuchangzao’ jujube demonstrated that the *BAM* gene was significantly upregulated during starch and sucrose metabolism [14]. A KEGG pathway analysis disclosed that BAM mainly regulates starch-maltose interconversion, starch hydrolysis to maltose, and maltose degradation to glucose. When large amounts of starch accumulate, BAM hydrolyzes starch into maltose and thence to glucose. It also directly hydrolyzes starch to glucose. The latter is then transformed to UDP-glucose which participates in sucrose formation. UDP-glucose enters different metabolic pathways including amino sugar and nucleotide sugar metabolism and glycolysis metabolism (Fig. 6a).

The transcriptome data for various treatment conditions were used to analyze jujube *BAM* gene expression levels (Tables S2 and S3). *ZjBAM* family members were clustered for the grafting (YJ) and root tiller (YG) propagation modes of the ‘Lingwuchangzao’ jujube fruit transcriptome [14]. The expression of *ZjBAM2* and *ZjBAM9* was substantially upregulated in both the propagation modes (Fig. 6b). *ZjBAM* family members were also classified for the fruit transcriptome at different developmental stages under elevated temperature and drought stress. The expression levels of *ZjBAM*4, *ZjBAM7*, *ZjBAM1*, and *ZjBAM9* were markedly lower than those of *ZjBAM*5, *ZjBAM6*, *ZjBAM3*, *ZjBAM2*, and *ZjBAM8* (Fig. 6c). The expression pattern analysis indicated that the *ZjBAM*2 and *ZjBAM*9 responded positively to sugar regulation under both ‘Lingwuchangzao’ jujube propagation modes, whereas *ZjBAM5*, *ZjBAM6*, *ZjBAM3*, *ZjBAM2*, and *ZjBAM8* responded to elevated temperature and drought stress.

### Expression patterns of *ZjBAM* genes under elevated temperature and drought stress

**Fig. 7:**
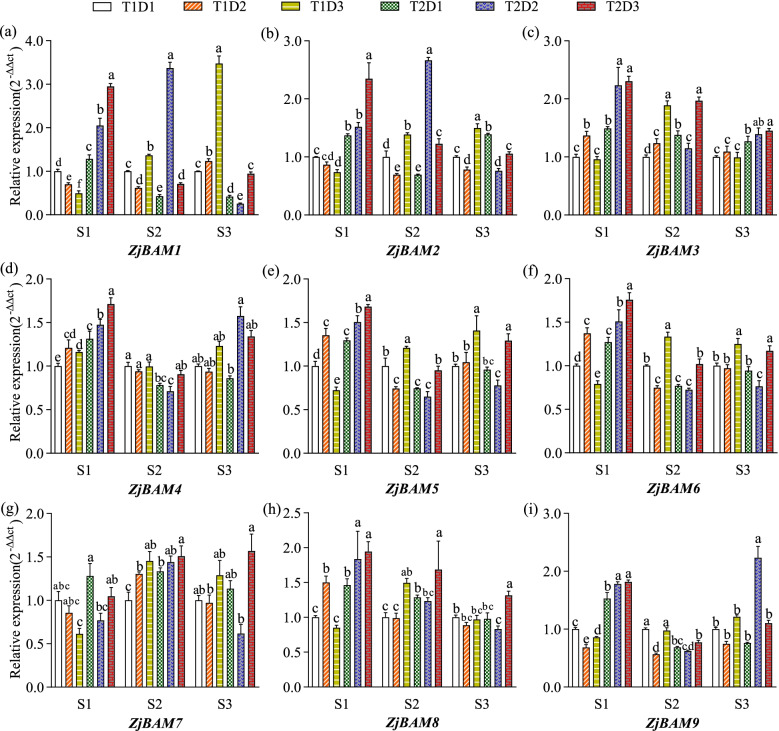
Relative expression of nine *ZjBAM* genes under abiotic stress (elevated temperature and drought) in three growth stages. Different lowercase letters denote significant difference (*p* < 0.05)

Based on the results of the transcriptome expression profiles analysis, nine *ZjBAM* genes were selected for quantitative reverse transcription-polymerase chain reaction (qRT-PCR) analysis at different jujube plant growth stages under elevated temperature and drought stress (Fig. 7). The expression patterns of the foregoing *ZjBAM* genes were similar at all three growth stages (S1, S2, and S3). In response to severe drought stress, expression of *ZjBAM1*, *ZjBAM2*, *ZjBAM5*, and *ZjBAM6* was significantly (*p* < 0.05) downregulated at S1 (T1D3) but significantly (*p* < 0.05) upregulated at S2 and S3 (Fig. 7a, b, e-g). In response to elevated temperature (T2D1), expression of *ZjBAM3* was significantly (*p* < 0.05) upregulated at all three growth stages, and that of *ZjBAM2* and *ZjBAM6* was significantly (*p* < 0.05) downregulated in S2 but significantly (*p* < 0.05) upregulated at S1 and S3 (Fig. 7b, f). Moreover, under T2D1, the expression of the *ZjBAM1*, *ZjBAM4*, and *ZjBAM9* was significantly (*p* < 0.05) upregulated at S1 but downregulated at S2 and S3 (Fig. 7a, d, i). Under T2D2, the expression of *ZjBAM3* was significantly (*p* < 0.05) upregulated at all three stages (Fig. 7c), whereas that of *ZjBAM5*, *ZjBAM6*, *ZjBAM8* was significantly (*p* < 0.05) upregulated only at S1 (Fig. 7e, f, h). However, under both elevated temperature and drought stress (T2D3), *ZjBAM3* and *ZjBAM8* expression levels increased significantly (*p* < 0.05) at all three growth stages (Fig. 7c, h), and *ZjBAM1*, *ZjBAM2*, *ZjBAM4*, *ZjBAM5*, *ZjBAM6*, and *ZjBAM9* were significantly (*p* < 0.05) upregulated at S1 (Fig. 7a, b, e, f, i). Hence, these nine ZjBAM gene family members responded positively to temperature, drought, and the interactive effects during jujube fruit development; they play a pivotal role in response to abiotic stress.

### Protein-protein interaction network analysis and validation of *ZjBAM* family genes

**Fig. 8:**
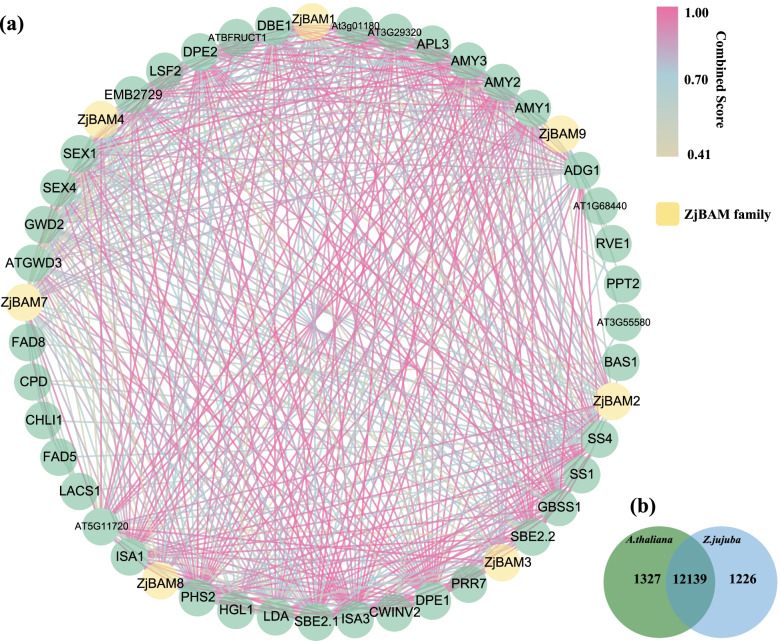
Interaction network of ZjBAM proteins. **a** Protein interaction network of ZjBAM family, yellow indicates jujube BAM family protein, green indicates interactor proteins. **b** Orthologous gene pairs between Jujube and *A. thaliana*

A network was constructed using the STRING database to investigate protein-protein interactions between ZjBAMs and *A.thaliana* proteins (Fig. 8a). A comparative genomic analysis of the jujube and *A.thaliana* genomes was performed with OrthoVenn2. A total of 12,139 genes pair orthologs were identified in jujube-*A. thaliana* (Fig. 8b). The *ZjBAM* family genes were used to retrieve the *BAM* genes among the *A.thaliana* orthologs. Seven *BAM* genes matched between the two plants (Table S4). Finally, a total of 47 proteins, including seven ZjBAM proteins, were identified at a medium confidence score of 0.400 (Table S4), and a protein-protein interaction network revealed their interactions (Fig. 8a). Furthermore, at a high confidence score (> 0.900), ZjBAM1, ZjBAM2, ZjBAM3, ZjBAM 4, ZjBAM7, and ZjBAM8 were found to interact with five, four, three, three, two, and four jujube proteins, respectively (Table S5). These results help elucidate the function of *ZjBAM* genes.

**Fig. 9:**
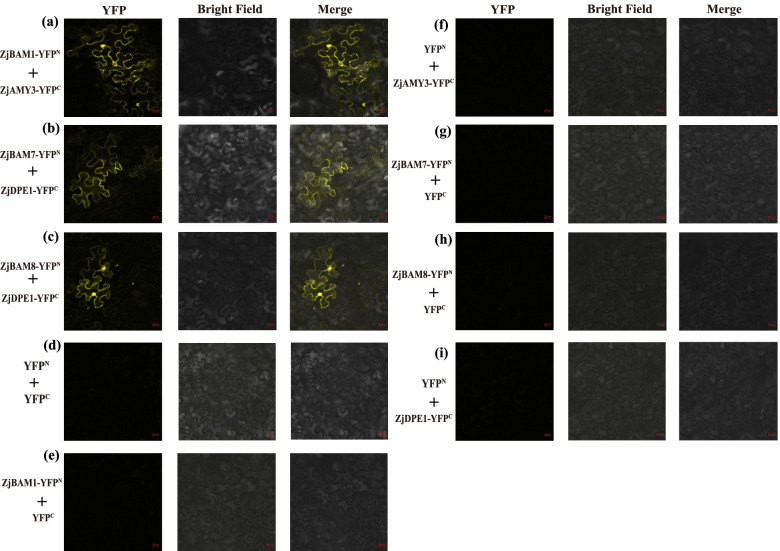
Validation of the ZjBAM protein interactions in tobacco based on BiFC assay. The yellow fluorescence signal of protein pairs of (**a**) ZjBAM1—ZjAMY3, **b** ZjBAM7—ZjDPE1, **c** ZjBAM8—ZjDPE1. **d** the control of yellow fluorescence protein (YFP) C-terminal and N-terminal. **e-i** The ZjBAM1, ZjAMY3, ZjBAM7, ZjDPE1, ZjBAM8 fused with YFP as a control, respectively

Furthermore, based on the functional annotation of seven jujube-*A. thaliana BAM* orthologs (Table S6), *ZjBAM1*-*CTBMY* is involved in cold resistance, regulates the accumulation of maltose and circadian regulation starch degradation. *ZjBAM7*-*BAM6* regulates in cellulose biosynthetic, carbohydrate metabolism, polysaccharide catabolism, and response to plant growth stages. *ZjBAM8*-*BMY2* which is repressed in the plant structure and growth stages, is involved in carbohydrate metabolism. Hence, these three genes of *ZjBAM1*, *ZjBAM7*, and *ZjBAM8* were selected as key genes for further protein interaction and functional analysis. Combined with the screening results with a high confidence interaction score (Table S5), three interaction proteins pairs—ZjBAM1 (Zj.jz015515046)-ZjAMY3 (Zj.jz040083023), ZjBAM7 (Zj.jz013313009)-ZjDPE1 (Zj.jz018223043), and ZjBAM8 (Zj.jz040841049)-ZjDPE1 (Zj.jz018223043)—were used to validate the protein-protein interactions by BiFC. The target genes, *ZjBAM1*, *ZjAMY3*, *ZjBAM7*, *ZjBAM8*, and *ZjDPE1* (Fig. S1a-e), were amplified using primers (Table S7). The *pCAMBIA1300YNE* plasmid was constructed and verified using double enzyme digestion, followed by sequencing (Shaanxi Breeding Biotechnologies Co., Ltd., Shaanxi, China) validation (Fig. S1f-j). Then, the recombinant plasmid was transformed into the *Agrobacterium* strain GV3101 and validated using colony PCR (Fig. S1k-o). Finally, the fusion plasmid constructs were generated and temporarily expressed in tobacco mesophyll cells to examine the yellow fluorescent signal. The results indicated that the ZjBAM1-ZjAMY3 (Fig. 9a, d, e, f) and ZjBAM8-ZjDPE1 (Fig. 9c, d, h, i) protein interactions were present in the plasma membrane and nucleus, whereas ZjBAM7-ZjDPE1 (Fig. 9b, d, g, i) interaction was present only in the plasma membrane. These findings were largely consistent with the predictions of the protein-protein interactions network analysis based on bioinformatics.

### Homology modeling of BAM in jujube

**Fig. 10:**
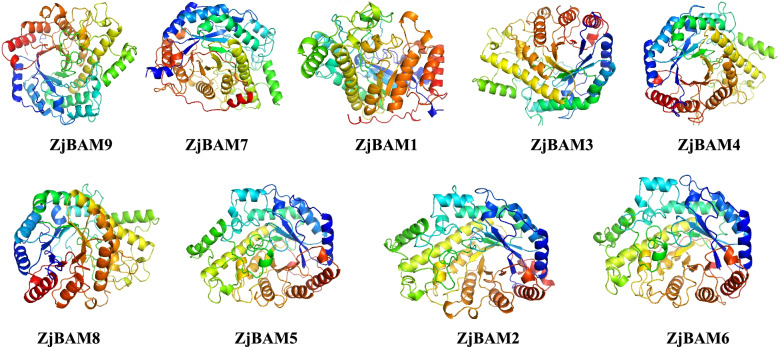
Tertiary structure of nine ZjBAM proteins

A 3D structure model of the nine jujube BAM family members was plotted using homology-based modeling (Fig. 10). Secondary protein structure predictions showed that all ZjBAM proteins were primarily composed of random coils (43.05–50.36%), followed by α-helices (30.89–39.28%), extended strands (11.84–15.66%), and β-turn (4.88–7.04%) (Table 1). No signal peptides or transmembrane regions were detected in any ZjBAM protein. The 3D structural quality was confirmed by calculating the root mean square deviation (RMSD). The RMSD for all models except that of ZjBAM2 was < 3 (ZjBAM1 = 1.732, ZjBAM3 = 1.941, ZjBAM4 = 1.631, ZjBAM5 = 2.101, ZjBAM6 = 2.101, ZjBAM7 = 0.326, ZjBAM8 = 1.276, ZjBAM9 = 2.970). Hence, the 3D protein structure predictions were reliable. Moreover, ZjBAM7 has an identical 3D structure model (RMSD = 0.326). The predicted 3D structure models of the ZjBAM protein family members suggest that their evolutionary paths and biological function are consistent.

## Discussion

The Chinese jujube is an economically important tree species with excellent abiotic stress resistance and rich nutritional composition. The metabolic conversion of sugars including glucose, fructose, and sucrose in fresh jujube fruits affects taste and market value [29]. The *BAM* genes control sugar degradation and conversion and play various regulatory roles in jujube development and abiotic stress response [30, 31]. *ZjBAM* genes directly or indirectly participate in starch, sucrose, glyoxylate, and dicarboxylate metabolism [14]. Herein, we identified nine *ZjBAM* genes in the jujube genome and comprehensively analyzed their structural characteristics, regulatory networks, abiotic stress responses, and protein interactions. As such, these findings establish a foundation for future studies investigating the functions of *BAM* genes and *BAM*-mediated sugar metabolism in jujube.

### Characterization and evolution of the *BAM* gene family in jujube

BAM (EC3.2.1.2) is an exohydrolase that acts on the non-reducing ends of linear α-1,4 glucosidic linkages. It successively cleaves the chains to produce maltose and limited dextrin [17, 32]. BAM belongs to the glycoside hydrolase family 14 and contains a typical Glyco_hydro_14 (PF01373) conserved domain at the *N*-terminus or a central position [33, 34]. *BAM* is a multigene family in several different plant species. Nine *BAM* genes were identified in *A. thaliana* [18], four in rice [35], nine in barley [36], 16 in banana [37], six in grapevine [31], 30 in *Brassica napus*, 11 in *B. rapa*, and 20 in *B. oleracea* [38]. Here, we identified nine genes (*ZjBAM1*–*ZjBAM9*) encoding BAM in the jujube genome database. This nine-gene subfamily was further divided into four groups classified by structural similarity. Conservation of the intron positions among the BAM family members of *Arabidopsis* indicated that there are two subfamilies in terrestrial plants. Subfamily I includes BAM1, BAM3, and BMA9, while subfamily II comprises BAM2, and BAM4–BAM8 [18, 39]. Multisequence alignment of the jujube BAM sequences revealed that Glu-186 and Glu-380 were highly conserved. The substrate-binding and active site of β-amylase contains the catalytic residues Glu-186 and Glu-380, as well as flexible loop and inner loops [19, 40]. Phylogenetic analysis of jujube, apple, poplar, cucumber, peach, and *A. thaliana* revealed that the BAM proteins were evolutionarily conserved. This discovery corroborated the results of BAM phylogenetic tree analyses of *A. thaliana*, peach, tomato, apple, citrus, strawberry, and pear [31]. The hydrolytic pathways of starch degradation generally involve AMY, SS, and BAM. AMY and SS are key carbohydrate hydrolytic enzymes that catalyze starch breakdown [33, 41, 42]. AMY, DPE, and ISA proteins that interact with ZjBAM proteins were identified by protein interaction network analysis. Furthermore, ZjBAM1-ZjAMY3, ZjBAM7-ZjDPE1, and ZjBAM8-ZjDPE1 protein interactions that were present in the plasma membrane and nucleus were validated by BiFC. The α-amylase (AMY1/3) are key carbohydrate hydrolytic enzymes that act synergistically with β-amylase toward efficient starch degradation [17]. 4-α-glucanotransferases (DPE1/2) are cytosolic proteins with transglucosidase and amylomaltase activities, which are essential for formation of sucrose from starch and cellular metabolism. Mutant studies on ISA1/3, which encodes an isoamylase-type debranching enzyme, show that the gene is involved in starch breakdown, whose mutation could cause the loss of detectable isoamylase activity, disruption of normal starch structure, reduced starch content, and abnormally structured amylopectins and phytoglycogens. Additionally, AT5G11720 is a hydrolase that is involved in carbohydrate metabolism and hydrolyzes O-glycosyl compounds; it is expressed in 23 plant structures during 13 growth stages. In addition, other interacting proteins, such as GBSS1 (Granule-bound starch synthase 1) and SS (starch synthase) are important enzymes regulating the catalytic degradation of starch and other sugars [42–44].

An evolutionary analysis of 961 BAM sequences in 136 species disclosed that *BAM* genes were extensively duplicated in terrestrial plants to increase the gene copy number and promote functionalization [19]. A single tandem gene pair was detected in the jujube *BAM* gene family and localized to chr 12 (Fig. 3a). Moreover, it had a Ka/Ks < 1.0, indicating that it may undergo strong purifying selection for retention. An intergenomic collinearity analysis identified five collinear *BAM* family gene pairs between peach and jujube, eight pairs between jujube and apple (Fig. 5a), five pairs between poplar and jujube, two pairs between jujube and *A. thaliana* (Fig. 5b), and three pairs between jujube and cucumber (Fig. 5c). This indicates that the duplicated genes might have diverged during evolution. Similar results were found in the syntenic relationships among the *BAM* genes of *Arabidopsis* and *Brassica* [38].

### Regulation of *BAM* genes in plant metabolism and abiotic stress response in jujube

Sugar metabolism plays a vital role in plant growth and development. Plants respond to abiotic stress by modulating carbohydrate conversion [45, 46]. Starch is a transitional form of carbon assimilation during fleshy fruit development. Its degradation is intimately associated with fruit ripening and metabolism [47, 48]. BAM modulates starch degradation, participates in plant sugar metabolism, and responds to abiotic stress [20]. Numerous *BAM* genes are involved in plant stress response. For instance, *AaBAM3.1* overexpression increases freezing tolerance in *A. thaliana*; heterologous *AaBAM3.1* overexpression yields consistent results [49]. Moreover, the expression of *BnaBAM3* and *BnaBAM5* is significantly upregulated in *Brassica* under heat stress [38]. Meanwhile, *VvBAM1* overexpression in tomatoes alters the soluble sugar levels and improves cold tolerance [31]. The expression of *CsBAM* gene family members is significantly upregulated in *Camellia sinensis* under ABA, salt, drought, and cold stress [17]. In jujube, expression of *BAM* has been reported as significantly upregulated in the sugar- and acid-related metabolic pathways in the transcriptomes of ‘Lingwuchangzao’ jujube propagated by grafting and root tillers [14]. Here, we analyzed the transcriptional profiles of nine *ZjBAM* genes in jujube subjected to elevated temperature and drought and found that the expression of *ZjBAM5*, *ZjBAM6*, *ZjBAM3*, *ZjBAM2*, and *ZjBAM8* were markedly upregulated. A qRT-PCR analysis showed that the expression of these genes was significantly (*p* < 0.05) upregulated in response to the interaction between elevated temperature and drought stress. Additionally, expression of *ZjBAM1*, *ZjBAM2*, *ZjBAM5,* and *ZjBAM6* was significantly upregulated in response to severe drought, while the expression of nine *ZjBAM* genes was significantly upregulated in response to high temperature stress.

We also found that *ZjBAM* genes contain several *cis*-acting elements, including the stress-related STRE, WRE3, and W, the phytohormone-related ARE, GARE-motif, MYC, ABRE, and ERE, as well as the growth and development-related AAGAA, MRE, MBSI, MYB, and O2-site. *ZjBAM3* was particularly enriched in stress-responsive elements (Fig. 3b), which corresponds to the significant expression in three growth stages under elevated temperature (T2D1), suggesting a potential regulatory function. For the *BAM* gene family members of *C. sinensis,* a stress-related *cis*-acting element has been reported, including ABRE, MBS, STRE, MYB, MYC, and ERE [17]. DRE, LTRE, and STRE respond to drought, low temperature, and heat stress in bread wheat [50]. Promoters may regulate the temporal and spatial expression of genes, and these *cis*-acting elements are important for gene function regulation. Consequently, further validation of the molecular regulatory mechanism of *ZjBAM* genes to abiotic stress is warranted in future studies.

As jujube trees have a long growth cycle, extremely high genomic heterozygosity, and challenges associated with transformation [1, 51], it would be feasible to clone the *ZjBAM* genes into a tomato by constructing an overexpression vector and determining the sugar content under elevated temperature and drought stress conditions, to clarify the direct regulatory relationship between *ZjBAM* and sugar content. Furthermore, constructs containing the stress-related promoter fused to the β-glucuronidase gene will allow for the identification of the core segment that responds to temperature and drought-induced expression. Additionally, a yeast one-hybrid system will facilitate the screening of key transcription factor proteins, which will require further verification by a dual-luciferase reporter gene assay. This system will further help elucidate regulatory mechanisms of *ZjBAM* genes expressed under elevated temperatures and the drought-induced regulation of jujube sugar metabolism, while assessing the effect of climate change on the quality of jujube fruit at the molecular level.

## Conclusions

The present study is a comprehensive analysis of the *ZjBAM* gene family in jujube. Nine *ZjBAM* genes were identified and divided into four groups. Their basic physicochemical characteristics, conserved domains, structures, motif compositions, *cis*-acting elements, chromosomal localizations, protein interaction networks, and 3D structure models were analyzed. Phylogenetic and synteny analyses of the *BAM* genes from various plant species provided novel perspectives regarding the evolution of the jujube *BAM* gene family. Regulatory and expression analyses demonstrated that jujube *BAM* genes control sugar metabolism and positively respond to elevated temperature and drought stress. Furthermore, the ZjBAM family interacting proteins were identified and a protein-protein interaction network was established. Three interaction proteins pairs ZjBAM1-ZjAMY3, ZjBAM7-ZjDPE1, and ZjBAM8-ZjDPE1 were verified to be present in the plasma membrane and nucleus by BiFC. Thus, collectively, this study provides a theoretical basis for further exploration of sugar metabolism responses to elevated temperature and drought stress, while providing candidate genes for future breeding of jujube with high sugar content and excellent resistance.

## Methods

### Identification of *ZjBAM* family genes

The jujube *BAM* (β-amylase, EC:3.2.1.2| gene 23,406) gene was first reported to be involved in the regulation of sugar and organic acid metabolism in the fruits of different propagations of ‘Lingwuchangzao’ jujube. Transcriptome differential analysis indicates that the *BAM* gene is significantly upregulated during sugar transformation; however, the background and gene family members of jujube remain unknown [14]. Here, we identified the *BAM* gene family members of the jujube genome. BAM protein sequence was elucidated with InterPro (https://www.ebi.ac.uk/interpro/). It contains a Glyco_hydro_14 (IPR001554, glycoside hydrolase family 14) conserved domain. A hidden Markov model (HMM) profile (PF01373.19) was obtained by searching the Pfam database (http://pfam.xfam.org/family/PF01373.19). HMMER3.1 identified *BAM* gene family members in the jujube genome (https://datadryad.org/stash/dataset/doi:10.5061/dryad.83fr7) [1]. The Expect (e) cutoff was set to 0.0001 (http://hmmer.org/). Nine *ZjBAM* gene family members were identified, and their Glyco_hydro_14 conserved domains were authenticated with SMART (http://smart.embl.de/) and the Conserved Domain Database (https://www.ncbi.nlm.nih.gov/cdd/). The gene family members were named *ZjBAM1*–*ZjBAM9*. Their physicochemical properties including molecular formulas and weights, isoelectric points (pI), instability and aliphatic indices, and grand averages of hydropathicity (GRAVY) were estimated with the ExPASy database (https://web.expasy.org/protparam/). Their protein secondary structures were predicted with SOPMA (https://npsa-prabi.ibcp.fr/NPSA/npsa_sopma.html). PSort II (http://psort.hgc.jp/) was used to predict the subcellular localization of the ZjBAM proteins.

### Conserved domain alignment, phylogenetic tree, exon-intron structure, and conserved motif analyses

The amino acid sequences of the *BAM* gene family member domains were aligned with DNAMAN v. 6.0.1 (Lynnon Biosoft, San Ramon, CA, USA). The phylogenetic tree of the *BAM* gene family members from jujube and other species was constructed using the neighbor-joining (NJ) method with 1000 bootstrap replications in MEGA X v. 10.1.1 (https://megasoftware.net). Based on an evolutionary scenario of genome rearrangements within the Rhamnaceae and on a possible gene family expansion and contraction in jujube and other species [1], apple (*M. domestica*), poplar (*P. trichocarpa*), cucumber (*C. sativus*), peach (*P. persica*), and *A. thaliana* species were selected to construct a phylogenetic tree. The genomic data of these species were downloaded from Ensembl (http://plants.ensembl.org/index.html). Exon-intron structures were analyzed with GSDS v. 2.0 (http://gsds.gao-lab.org/). The conserved motif was identified with MEME (https://meme-suite.org/meme/). TBtools (https://github.com/CJ-Chen/TBtools) was used to visualize the phylogenetic tree of the gene family, conserved domain, and motifs [52].

### Chromosome localization, gene duplication, *cis*-acting element, and synteny analyses

The *ZjBAM* gene members were localized using the gene annotations in GFF files and plotted and visualized with Mapchart v. 2.3 (https://www.wur.nl/en/show/Mapchart.htm). The loci of the *ZjBAM* genes on the genome and the regions 1500 bp upstream from them were selected as promoter regions used to predict the *cis*-acting elements in the PlantCARE (http://bioinformatics.psb.ugent.be/webtools/plantcare/html/) database. They were then visualized with GSDS v. 2.0 (gsds.gao-lab.org). MCScanX (https://github.com/wyp1125/MCScanx) was used to analyze gene duplication and collinearity [53]. Circos (http://www.circos.ca) was used to visualize genome collinearity. Nonsynonymous (Ka) and synonymous (Ks) substitution rates for gene pairs were evaluated with KaKs Calculator v. 1.2 (https://sourceforge.net/projects/kakscalculator2/). The Ks values were used to calculate the divergence time according to Eq. 1 [54, 55]:

1 $$\mathrm{T}=\mathrm{Ks}/2\mathrm{r}$$

where r = 1.5 × 10^− 8^ for dicotyledons.

### Plant material and abiotic stress treatments

**Fig. 11:**
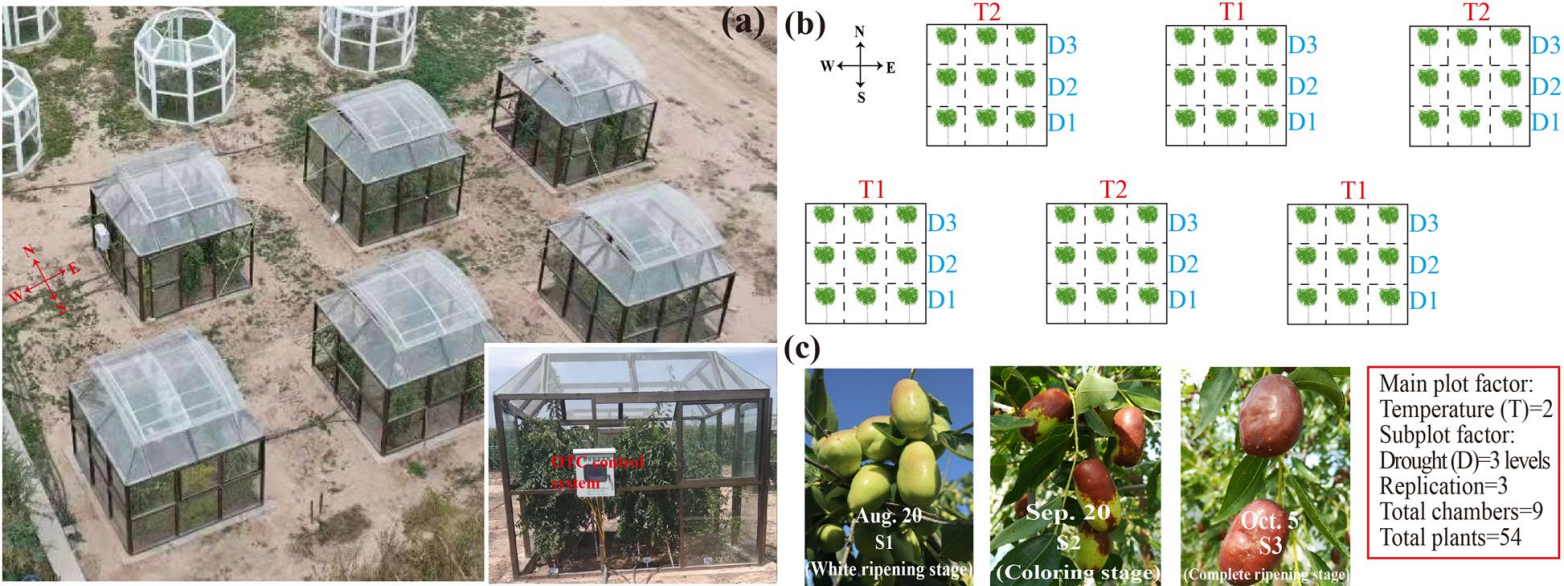
Experimental design, layout, and growth stages of jujube. **a** Open-top chambers (OTC). **b** Experimental design and layout. **c** Three growth stages of jujube fruit

‘Lingwuchangzao’ jujube fruits were obtained from the experimental farm of Ningxia University (38.78° N, 106.07° E, 1117 m a.s.l.). A split-plot design for a two-factor experiment was used with three replications (Fig. 11a and b). The main plot factor was air temperature (T) with two treatment levels: T1, natural air temperature; T2, elevated air temperature = T1 + (2 ± 0.5 °C). The subplot factor was drought (D), with three treatment levels: D1, 70–75% field capacity; D2, 50–55% field capacity; and D3, 30–35% field capacity. Each subplot contained three replicates. A total of 54 jujube trees (5-year-old) were planted in six open-top chambers (OTCs) (Fig. 11a and b, Table S8) [12]. The experiments were conducted in the OTC, a square with a length of 3.0 m and a height of 2.3 m, and each chamber was divided into nine subplots of 1.0 m × 1.0 m with an insulation plate. An OTC simulation control system was used to regulate and monitor soil moisture and air temperature, and an automatic solar irrigation system was used to control the soil moisture. Data were collected using multi-channel wireless data acquisition equipment (ZWSN-C-A) to monitor the atmospheric temperature and soil moisture in the container (Fig. 11a) [12]. The orientation of all plots was in a north-south configuration. The average tree height was 1.5 m, and the plants were spaced in a 1.0 m × 1.0 m arrangement. Previous studies showed that under elevated temperature and drought stress, the fruit’s physiological indicators, such as sugar, organic acid, pigments, and enzyme activities, are significantly altered, and differentially expressed genes are significantly enriched in the three growth stages of the fruit: white ripening, coloring, and complete ripening [12, 13, 56, 57]. Samples were collected according to previously reported methods [12, 13]. Approximately 20 fruit samples from each tree were collected from marked fruit clusters during the fruit white ripening (S1; 08/20/2020), coloring (S2; 09/20/2020), and complete ripening stages (S3; 10/05/2020) to ensure that the collected fruits had the same flowering time and fruit development stage (jujube has an indefinite inflorescence) (Fig. 11c). Each treatment consisted of three replicates (three trees), and 20 fruits were collected from each tree and mixed for subsequent experiments. The samples were collected in liquid nitrogen and stored at − 80 °C until transcriptome profiling and qRT-PCR analyses. Each treatment contained three biological replicates that were used for Illumina sequencing and qRT-PCR analysis, and each was performed in three technical repeats, respectively.

### RNA-seq analysis

Total RNA was extracted from the jujube fruit samples using an OmniPlant RNA Kit (DNase I) (CWBIO, Beijing, China). following the manufacturer’s instructions. RNA quality was assessed using 2% gel electrophoresis. RNA concentration was determined with a NanoDrop 2000 spectrophotometer (Thermo Fisher Scientific, Waltham, MA, USA). The cDNA libraries were constructed with an Illumina® TruSeqTM RNA Sample Preparation Kit (Illumina Inc., San Diego, CA, USA) and an Illumina® Novaseq 6000 System (Shanghai Meiji Biological Medicine Technology Co., Ltd., Shanghai, China). After sequencing, the raw reads were filtered, and clean data were obtained by removing low-quality reads and adapter sequences. High-quality clean data were aligned to the reference genome sequence of jujube with HISAT2 v. 2.0.1 (http://daehwankimlab.github.io/hisat2/download/) [1, 51]. Read count data were used to analyze differential gene expression levels in the EdgeR package in R version 3.3.1 (R Core Team, Vienna, Austria) [58].

### Metabolic pathway regulation and expression profile analysis

The protein sequences of all *ZjBAM* gene family members were submitted to metabolic pathway mapping using the KEGG pathway database BlastKOALA (https://www.kegg.jp/blastkoala/). The *ZjBAM* gene family member expression profiles were analyzed based on the transcriptome data for grafting and root tiller propagation [14] as well as those for elevated temperature and drought stress. The gene expression levels were calculated using the fragments per kilobase per million (FPKM) values. Gene expression was visualized using the heatmap package in R version 3.3.1.

### qRT-PCR and statistical analysis

The *ZjBAM* family gene expression patterns were analyzed using qRT-PCR in a Lightcycler 480 System (Roche Diagnostics, Basel, Switzerland). The primers were designed according to the full cDNA sequence (Table S7) using PrimerQuest (http://sg.idtdna.com/Primerquest/Home/Index). *CYP* and *ACTIN9* were the internal reference genes [59]. First-strand reverse transcription was performed with a HiScript® II Q RT SuperMix for qPCR (+gDNA Wiper) Kit (R223–01; Vazyme Biotech, Nanjing, China). Four microliters of 4x gDNA Wiper Mix, total RNA, and RNase-free ddH_2_O were combined to constitute a final volume of 16.0 μL and used to remove genomic DNA. Then, 4.0 μL 5x HiScript II qRT SuperMix II was added to produce a final 20.0 μL for reverse transcription. The program was the following: 50 °C for 15 min, 85 °C for 5 s to inactivate the enzyme, and temperature decrease to 4 °C. Then, qRT-PCR was performed using a ChamQ Universal SYBR qPCR Master Mix Kit (Vazyme Biotech, Nanjing, China). The reaction system volume was 20 μL and consisted of 10 μL of 2x ChamQ Universal SYBR qPCR Master Mix, 0.4 μL PCR forward and reverse primers (10 μM), 1.0 μL cDNA template, and 8.2 μL ddH_2_O. The reaction consisted of denaturation at 95 °C for 5 min followed by 40 cycles of 95 °C for 30 s and 60 °C for 1 min. For each PCR, there were at least three biological and technical replicates. Relative *ZjBAM* expression levels were calculated using the 2^−ΔΔCt^ method [60, 61]. Statistical analysis consisted of one-way ANOVA and was conducted in GraphPad Prism v. 8 (GraphPad Software Inc., La Jolla, CA, USA). Tukey’s HSD multiple comparisons were applied at the 0.05 significance level.

### Gene interaction network of ZjBAM proteins

As *A. thaliana* is a model plant, an accurate and complete record of its protein interaction network is available in the STRING database (https://string-db.org/). Hence, in this study, orthologous genes were identified by comparing the jujube and *A. thaliana* genomes and subsequently mapping the jujube BAM protein interaction network through the *A. thaliana* protein interaction network. Firstly, jujube genome protein sequences were submitted to OrthoVenn2 (https://orthovenn2.bioinfotoolkits.net/home) for comparative genome analysis with *A. thaliana* to obtain all orthologous pairs between jujube and *A. thaliana*. Secondly, the ZjBAM protein interaction network was constructed with the STRING database based on *A. thaliana* and jujube orthologous pairs. Gene interactions between *A. thaliana* and jujube were identified and visualized using Cytoscape v. 3.8.2 (https://cytoscape.org/download.html).

### Gene cloning, vector construction, and BiFC

The target gene sequences of ZjBAM1 (Zj.jz015515046), ZjAMY3 (Zj.jz040083023), ZjBAM7 (Zj.jz013313009), ZjBAM8 (Zj.jz040841049), and ZjDPE1 (Zj.jz018223043) were extracted from the jujube genome sequences and subsequently cloned by PCR. Primers were designed using Primer Premier 6.0 software (Premier Biosoft, CA, Palo Alto, CA, USA) (Table S7). The reaction mixture for PCR amplification contained the following: 20.0 μL 2x Phanta® Max Master Mix (P515, Vazyme, Nanjing, China), 1.0 μL each of forward and reverse primers, 0.5 μL cDNA template, and 17.5 μL RNase-free ddH_2_O, thus obtaining a final volume of 40.0 μL. The PCR reaction was performed as follows: pre-denaturation at 95 °C for 10 min, followed by 30 cycles of 95 °C for 30 s, 58 °C for 30 s, 72 °C for 30 s, and a final extension at 72 °C for 10 min. Products of PCR were visualized and verified using 1% agarose gel electrophoresis. The products were then extracted and purified using the HiPure Gel Pure DNA Mini Kit (Magen, Shanghai, China) and stored at − 20 °C. The plasmids were driven by the 35S promoter of *pCAMBIA1300YNE* and were digested with Xba I and Kpn I; a gel recovery kit (Bioteke, Beijing, China) was used to recover the enzyme cut products. The double-enzyme digestion reaction was performed using the following mixture: 4.0 μL 1x Tango buffer, 2.0 μL Xba I, 1.0 μL Kpn I, 4.0 μg plasmids, and approximately 40.0 μL of ddH_2_O. Samples were incubated at 37 °C for 40 min, followed by 65 °C for 15 min until enzyme inactivation. The target DNA fragment and linearized carrier were added to the centrifugal tube with a certain molar ratio (3:1–10:1) for recombination reaction. After mixing at 50 °C for 15 min, there was an immediate transformation, and the remaining sample was stored at − 20 °C.

Transformation and positive cloning selection: a tube containing 50.0 μL of DH5α competent cells was placed on melting ice and lightly shaken to suspend the cells. Then, 10.0 μL of reaction fluid was added. This solution was shaken lightly and incubated for 30 min on ice. The solution was then placed in a 42 °C water bath for 45 s to initiate heat shock, after which it was quickly placed on ice for 2 min. Then, 700 μL of LB liquid medium was added, followed by incubation at 37 °C for 60 min. The sample was centrifuged at 5000 rpm for 2 min to obtain the bacteria. Then, 400 μL of bacteria were spread evenly on an agar plate containing Kanamycin, followed by coating with sterilized glass beads. After the bacterial solution was absorbed by the agar, it was inverted and maintained at 37 °C overnight. The colonies were selected for cloning and identification by PCR.

*Agrobacterium* strain GV3101 transformation: Plasmid Mini Kit I (D6943–01, Omega Bio-Tek, Beijing, China) was used to extract the right sequencing plasmid. The procedure was as follows: GV3101 competent cells were placed on ice and 5.0–10.0 μL of plasmid was added, followed by an ice bath for 15 min; then, they were frozen in liquid nitrogen for 5 min, and thereafter, placed in a 37 °C water bath for 5 min, and then in an ice bath for 5 min. Then, 700 μL of antibiotic-free LB liquid medium was added to the mixture, followed by shaking at 28 °C for 2 h, and centrifugation at 5000 rpm for 1 min. The bacterial solution (400 μL) was spread on a plate (containing 50 mg·mL^− 1^ kanamycin and 25 mg·mL^− 1^ rifampicin), which was inverted and maintained at 28 °C. After the formation of a single colony, six single colonies per gene were selected for colony PCR verification. The primers were the same as those used for amplification.

Transient transformation of tobacco to perform BiFC: For BiFC assays, the coding regions of *ZjAMY3* and *ZjDPE1* were ligated into *pCAMBIA1300YNE* and fused with the C-terminus of yellow fluorescence protein (YFP). *ZjBAM1*, *ZjBAM7*, and *ZjBAM8* were cloned into *pCAMBIA1300YNE* and fused with the N-terminus of YFP. The recombinant constructs were co-transformed in pairs into tobacco leaves, and the fluorescence signal was examined using a Leica TCS SP8 laser confocal microscope (Leica, Germany). The transformation was performed as follows: the tested *Agrobacterium* strain GV3101 solution was centrifuged (200 rpm) at 28 °C overnight. Then, 1–1.5 mL of bacterial solution was added to a sterile centrifuge tube to perform centrifugation at 1000 rpm for 10 min. The bacterial solution precipitated at room temperature (20 °C); the supernatant was discarded and suspended the bacteria. This step was repeated to further remove the remaining antibiotics. A small amount of suspended bacteria solution was diluted 10 times, followed by OD_600_ measurement. The solution was added to a 1.5 mL centrifuge tube and maintained at 20 °C for 1–3 hours to initiate infection. Before infection, the tobacco samples were placed under a white fluorescent lamp for 1 h to open pores. A suspension of 1.0 mL was injected into tobacco leaves using a plastic syringe. Then, the plants were cultured overnight in the dark and transferred to a culture room for 2 d. The infection area was cut, and the epidermis was peeled under a fluorescence microscope.

### Homology modeling of ZjBAM 3D structure

Homology modeling was used to predict the 3D structure of the ZjBAM gene family members. The most similar template was selected using PSI-BLAST against the PDB database (https://www.rcsb.org/). The protein tertiary structures were modeled with the homology modeling server SWISS-MODEL (https://swissmodel.expasy.org/). Model quality was assessed using the SAVES server (https://servicesn.mbi.ucla.edu/) and further analyzed and refined with PyMOL v. 1.7.4 (https://osdn.net/projects/sfnet_pymol/downloads/pymol/1.7/pymol-v1.7.4.0.tar.bz2/).

## Supplementary Information

**Additional file 1: Table S1.** Collinear gene pairs of *BAM*-encoding genes among *Z. jujuba*, *Malus domestica*, *Prunus persica*, *Arabidopsis thaliana*, *Populus trichocarpa*, and *Cucumis sativus*.

**Additional file 2: Table S2.** Statistics of the transcriptome data of ‘Lingwuchangzao’ jujube under temperature and drought stress.

**Additional file 3: Table S3.** Transcriptome FPKM expression data of grafting (YJ) and root tiller (YG) propagation of ‘Lingwuchangzao’ jujube subjected to elevated temperature and drought conditions.

**Additional file 4: Table S4.** Protein interactions of members of ZjBAM family and their corresponding orthologs in *A. thaliana*.

**Additional file 5: Table S5.** ZjBAM family protein interactions at high confidence (0.900).

**Additional file 6: Table S6.** Annotation of ZjBAM family and corresponding orthologs in *A. thaliana*.

**Additional file 7: Table S7.** List of primer sequences used in this study.

**Additional file 8: Table S8.** The experimental design: a split-plot design for two-factor treatments.

**Additional file 9: Figure S1.** Amplification of target genes, vector identification, and transformation of *Agrobacterium* strain GV3101 followed by PCR identification.

## Data Availability

All data and materials used in this study are publicly available. The nine *ZjBAM* gene sequences and *Ziziphus jujuba* Mill. genome sequence information was obtained from the website https://datadryad.org/stash/dataset/doi:10.5061/dryad.83fr7. Genomic data for apple (*Malus domestica*), poplar (*Populus trichocarpa*), cucumber (*Cucumis sativus*), peach (*Prunus persica*), and *Arabidopsis thaliana* were obtained from Ensembl (http://plants.ensembl.org/index.html). All data generated or analyzed during this study are included in this published article and its supplementary information files.

## Abbreviations

BAM: *β*-amylase
TCM: Traditional Chinese medicine
HMM: Hidden Markov model
GRAVY: Grand averages of hydropathicity
Glyco_hydro_14: Glycoside hydrolase family 14
pI: Isoelectric points
NJ: Neighbor-joining
Ka: Nonsynonymous
Ks: Synonymous
OTCs: Open-top chambers
qRT-PCR: Quantitative real-time polymerase chain reaction
BiFC: Bimolecular fluorescence complementation
YFP: Yellow fluorescence protein
