# Supplementary material for: Genome-wide identification of BAM (β-amylase) gene family in jujube (Ziziphus jujuba Mill.) and expression in response to abiotic stress
Source: BMC Genomics. 2022 Jun 13;23:438. doi: 10.1186/s12864-022-08630-5 (PMC9195466; doi:10.1186/s12864-022-08630-5)
Supplement: Supplementary file 1 — Additional file 1: Table S1. Collinear gene pairs of BAM-encoding genes among Z. jujuba, Malus domestica, Prunus persica, Arabidopsis thaliana, Populus trichocarpa, and Cucumis sativus. [file 12864_2022_8630_MOESM1_ESM.docx]

| **Table S1** **Collinear gene pairs of *BAM*-encoding genes** **among** ***Z. jujuba*,** ***Malus domestica*,** ***Prunus persica*,** ***Arabidopsis thaliana*,** ***Populus trichocarpa*, and** ***Cucumis sativus*.** | | | | | | | | | | |
| --- | --- | --- | --- | --- | --- | --- | --- | --- | --- | --- |
| ***Ziziphus jujuba*** | ***Populus trichocarpa*** | **Score**  **(direction)** | ***Malus domestica*** | **Score**  **(direction)** | ***Arabidopsis thaliana*** | **Score**  **(direction)** | ***Prunus persica*** | **Score**  **(direction)** | ***Cucumis sativus*** | **Score**  **(direction)** |
| *ZjBAM1* | PNT54600  PNT44409 | 1530(+)  1440(+) | MD04G0051300  MD06G0036900 | 1480(+)  1450(+) |  |  | ONI06287 | 1500(+) | KGN55808 | 1230(+) |
| *ZjBAM2* |  |  | MD13G0198500  MD16G0199600 | 1330(+)  1390(+) | AT3G23920 | 1140(+) | ONI26897 | 1430(+) | KGN56795 | 1390(+) |
| *ZjBAM3* |  |  |  |  |  |  |  |  |  |  |
| *ZjBAM4* |  |  |  |  |  |  |  |  |  |  |
| *ZjBAM5* | PNT53474  PNT45580 | 1110(+)  1030(+) | MD14G0115100  MD06G0090200 | 1320(+)  1320(+) |  |  | ONI07524 | 1280(+) |  |  |
| *ZjBAM6* |  |  |  |  |  |  |  |  |  |  |
| *ZjBAM7* | PNT95151 | 1320(+) | MD09G0090300 | 1560(+) | AT2G32290 | 848(+) | ONI18541 | 1710(+) | KGN43548 | 1270(+) |
| *ZjBAM8* |  |  |  |  |  |  |  |  |  |  |
| *ZjBAM9* |  |  | MD13G0153700 | 1240(+) |  |  | ONI28432 | 1320(+) |  |  |
