# Supplementary material for: Genome-wide identification of BAM (β-amylase) gene family in jujube (Ziziphus jujuba Mill.) and expression in response to abiotic stress
Source: BMC Genomics. 2022 Jun 13;23:438. doi: 10.1186/s12864-022-08630-5 (PMC9195466; doi:10.1186/s12864-022-08630-5)
Supplement: Supplementary file 2 — Additional file 2: Table S2. Statistics of the transcriptome data of ‘Lingwuchangzao’ jujube under temperature and drought stress. [file 12864_2022_8630_MOESM2_ESM.docx]

| **Table S2 Statistics of the transcriptome data of ‘Lingwuchangzao’ jujube under temperature and drought stress.** | | | | | | | |
| --- | --- | --- | --- | --- | --- | --- | --- |
| Sample | Total reads | Total bases | Total_Reads_with_Ns | Error% | Q20% | Q30% | GC% |
| S1_T1D1 | 43361322 | 6547559622 | 168950 | 0.0298 | 96.15 | 89.95 | 44.55 |
| S1_T1D2 | 44334076 | 6694445476 | 180907 | 0.0305 | 95.88 | 89.42 | 44.91 |
| S1_T1D3 | 47682846 | 7200109746 | 195401 | 0.0294 | 96.31 | 90.28 | 44.79 |
| S1_T2D1 | 51623590 | 7795162090 | 211065 | 0.0296 | 96.18 | 90.15 | 45.18 |
| S1_T2D2 | 51533622 | 7781576922 | 211038 | 0.0309 | 95.72 | 89.16 | 45.27 |
| S1_T2D3 | 50609102 | 7641974402 | 208574 | 0.0301 | 96.02 | 89.84 | 45.24 |
| S2_T1D1 | 46554210 | 7029685710 | 190548 | 0.0312 | 95.65 | 88.94 | 44.95 |
| S2_T1D2 | 52030354 | 7856583454 | 212715 | 0.0296 | 96.25 | 90.07 | 44.93 |
| S2_T1D3 | 51654364 | 7799808964 | 210979 | 0.0306 | 95.88 | 89.39 | 45.11 |
| S2_T2D1 | 50853024 | 7678806624 | 208171 | 0.0303 | 95.96 | 89.63 | 45.18 |
| S2_T2D2 | 53993906 | 8153079806 | 221723 | 0.0295 | 96.3 | 90.24 | 45.09 |
| S2_T2D3 | 47319764 | 7145284364 | 192788 | 0.0296 | 96.22 | 90.22 | 45.41 |
| S3_T1D1 | 50722082 | 7659034382 | 208608 | 0.0305 | 95.93 | 89.45 | 44.79 |
| S3_T1D2 | 51141688 | 7722394888 | 209367 | 0.0302 | 95.97 | 89.7 | 45.31 |
| S3_T1D3 | 52054146 | 7860176046 | 213001 | 0.0311 | 95.64 | 89.02 | 45.07 |
| S3_T2D1 | 46048560 | 6953332560 | 189555 | 0.03 | 96.11 | 89.85 | 45.06 |
| S3_T2D2 | 49510086 | 7476022986 | 212696 | 0.0294 | 96.3 | 90.28 | 46.18 |
| S3_T2D3 | 50832878 | 7675764578 | 209428 | 0.0295 | 96.26 | 90.19 | 44.95 |
