# Supplementary material for: Genome-wide identification of BAM (β-amylase) gene family in jujube (Ziziphus jujuba Mill.) and expression in response to abiotic stress
Source: BMC Genomics. 2022 Jun 13;23:438. doi: 10.1186/s12864-022-08630-5 (PMC9195466; doi:10.1186/s12864-022-08630-5)
Supplement: Supplementary file 3 — Additional file 3: Table S3. Transcriptome FPKM expression data of grafting (YJ) and root tiller (YG) propagation of ‘Lingwuchangzao’ jujube subjected to elevated temperature and drought conditions. [file 12864_2022_8630_MOESM3_ESM.docx]

| **Table S3 Transcriptome FPKM expression data of grafting (YJ) and root tiller (YG) propagation of ‘Lingwuchangzao’ jujube subjected to elevated temperature and drought conditions.** | | | | | | | | | | | | | | | | | | | | | | | | |
| --- | --- | --- | --- | --- | --- | --- | --- | --- | --- | --- | --- | --- | --- | --- | --- | --- | --- | --- | --- | --- | --- | --- | --- | --- |
| Gene | YJ1 | YJ2 | YJ3 | YG1 | YG2 | YG3 | S1_T1D1 | S1_T1D2 | S1_T1D3 | S1_T2D1 | S1_T2D2 | S1_T2D3 | S2_T1D1 | S2_T1D2 | S2_T1D3 | S2_T2D1 | S2_T2D2 | S2_T2D3 | S3_T1D1 | S3_T1D2 | S3_T1D3 | S3_T2D1 | S3_T2D2 | S3_T2D3 |
| ZjBAM1 | 14.94904 | 17.20482 | 16.50114 | 7.20557 | 7.031869 | 5.41716 | 2.46 | 2.13 | 1.14 | 1.16 | 3.02 | 1.35 | 0 | 0.17 | 0.03 | 0.1 | 0.13 | 0.82 | 0.34 | 0.17 | 0.2 | 0.45 | 0.25 | 0.24 |
| ZjBAM2 | 189.9258 | 211.8399 | 282.7554 | 231.2838 | 212.4235 | 375.2273 | 17.07 | 16.91 | 17.38 | 17.66 | 17.69 | 22.31 | 16.65 | 15.22 | 13.57 | 14.97 | 20.77 | 17.18 | 13.53 | 9.14 | 11.09 | 15.68 | 11.06 | 10.33 |
| ZjBAM3 | 2.918282 | 5.852825 | 5.61306 | 5.677242 | 5.249619 | 2.840431 | 11.29 | 18.31 | 21.15 | 10.5 | 14.28 | 8.28 | 3.13 | 2.57 | 5.28 | 3.78 | 3.41 | 4.63 | 11.9 | 10.99 | 6.49 | 12.91 | 8.96 | 15.17 |
| ZjBAM4 | 9.801912 | 9.370208 | 10.9802 | 11.72792 | 7.635774 | 9.546035 | 0 | 0 | 0 | 0 | 0 | 0 | 0 | 0 | 0 | 0 | 0 | 0 | 0 | 0 | 0 | 0 | 0 | 0 |
| ZjBAM5 | 17.38624 | 16.5044 | 19.97259 | 23.33175 | 21.95459 | 25.6446 | 32.32 | 47.25 | 19.03 | 24.54 | 41.62 | 22.06 | 1.58 | 2.11 | 0.44 | 1.45 | 3.95 | 16.32 | 4.8 | 2.86 | 5.73 | 8.2 | 7.11 | 2.77 |
| ZjBAM6 | 17.38624 | 16.5044 | 19.97259 | 23.33175 | 21.95459 | 25.6446 | 55.66 | 69.96 | 43.04 | 46.58 | 79.72 | 41.5 | 1.52 | 2.99 | 1.32 | 4.43 | 13.47 | 29.18 | 13.31 | 6.08 | 11.11 | 14.07 | 12.47 | 5.22 |
| ZjBAM7 | 10.5401 | 29.14809 | 17.30999 | 21.67647 | 8.477718 | 4.820064 | 0 | 0 | 0 | 0 | 0 | 0 | 0 | 0 | 0 | 0 | 0 | 0 | 0 | 0 | 0 | 0 | 0 | 0 |
| ZjBAM8 | 11.11536 | 8.14958 | 9.456286 | 7.042474 | 9.320129 | 8.586378 | 13.79 | 12.37 | 13 | 14.93 | 12.87 | 11.09 | 20.48 | 15.91 | 19.87 | 19.09 | 18.75 | 8.52 | 15.07 | 14.94 | 17.85 | 13.09 | 17.36 | 15.81 |
| ZjBAM9 | 70.51931 | 70.02557 | 93.12913 | 66.53041 | 84.09572 | 104.6871 | 2.12 | 0.76 | 0.85 | 1.01 | 1.76 | 1.15 | 0 | 0 | 0.25 | 0 | 0.04 | 0.04 | 0.12 | 0.07 | 0 | 0.13 | 0 | 0 |
