# Supplementary material for: Genome-wide identification of BAM (β-amylase) gene family in jujube (Ziziphus jujuba Mill.) and expression in response to abiotic stress
Source: BMC Genomics. 2022 Jun 13;23:438. doi: 10.1186/s12864-022-08630-5 (PMC9195466; doi:10.1186/s12864-022-08630-5)
Supplement: Supplementary file 4 — Additional file 4: Table S4. Protein interactions of members of ZjBAM family and their corresponding orthologs in A. thaliana. [file 12864_2022_8630_MOESM4_ESM.docx]

| **Table S4 Protein interactions of members of ZjBAM family and their corresponding orthologs in *A. thaliana*** | | | |
| --- | --- | --- | --- |
| Gene name | Jujuba | *A.thaliana* | Id name |
|  | Zj.jz027983070 | AT5G48300 | ADG1 |
|  | Zj.jz006119172 | AT4G25000 | AMY1 |
|  | Zj.jz034129044 | AT1G76130 | AMY2 |
|  | Zj.jz040083023 | AT1G69830 | AMY3 |
|  | Zj.jz044781026 | AT4G39210 | APL3 |
|  | Zj.jz028947036 | AT1G68440 | AT1G68440 |
|  | Zj.jz042921011 | AT3G29320 | AT3G29320 |
|  | Zj.jz044313110 | AT3G55580 | AT3G55580 |
|  | Zj.jz017401047 | AT5G11720 | AT5G11720 |
|  | Zj.jz009017010 | AT3G13790 | ATBFRUCT1 |
|  | Zj.jz019149007 | AT5G26570 | ATGWD3 |
|  | Zj.jz042921076 | AT3G01180 | At3g01180 |
| *ZjBAM7* | Zj.jz013313009 | AT2G32290 | BAM6 |
| *ZjBAM4* | Zj.jz029235021 | AT2G45880 | BAM7 |
|  | Zj.jz003705117 | AT2G26710 | BAS1 |
| *ZjBAM8* | Zj.jz040841049 | AT5G45300 | BMY2 |
| *ZjBAM9* | Zj.jz004069034 | AT5G18670 | BMY3 |
| *ZjBAM3* | Zj.jz029235020 | AT4G00490 | BMY9 |
|  | Zj.jz005267083 | AT4G18480 | CHLI1 |
|  | Zj.jz040731042 | AT5G05690 | CPD |
| *ZjBAM1* | Zj.jz015515046 | AT4G17090 | CT-BMY |
|  | Zj.jz043343196 | AT3G52600 | CWINV2 |
|  | Zj.jz001627042 | AT1G03310 | DBE1 |
|  | Zj.jz018223043 | AT5G64860 | DPE1 |
|  | Zj.jz003639090 | AT2G40840 | DPE2 |
|  | Zj.jz019719008 | AT3G20440 | EMB2729 |
|  | Zj.jz041763047 | AT3G15850 | FAD5 |
|  | Zj.jz040731063 | AT5G05580 | FAD8 |
|  | Zj.jz040945025 | AT1G32900 | GBSS1 |
|  | Zj.jz021445094 | AT4G24450 | GWD2 |
|  | Zj.jz025819170 | AT3G23640 | HGL1 |
|  | Zj.jz000817059 | AT2G39930 | ISA1 |
|  | Zj.jz039989054 | AT4G09020 | ISA3 |
|  | Zj.jz001627322 | AT2G47240 | LACS1 |
|  | Zj.jz044313044 | AT5G04360 | LDA |
|  | Zj.jz036321124 | AT3G10940 | LSF2 |
|  | Zj.jz008005085 | AT3G46970 | PHS2 |
|  | Zj.jz044875001 | AT5G02810 | PRR7 |
|  | Zj.jz043343271 | AT5G03650 | SBE2.1 |
|  | Zj.jz043343271 | AT5G03650 | SBE2.2 |
|  | Zj.jz021445094 | AT4G24450 | SEX1 |
|  | Zj.jz024411014 | AT3G52180 | SEX4 |
|  | Zj.jz036789115 | AT5G24300 | SS1 |
|  | Zj.jz042571069 | AT4G18240 | SS4 |
|  | Zj.jz002249057 | AT3G01550 | PPT2 |
| *ZjBAM2* | Zj.jz044849113 | AT3G23920 | TRBAMY |
|  | Zj.jz041823025 | AT5G17300 | RVE1 |
