# Supplementary material for: Genome-wide identification of BAM (β-amylase) gene family in jujube (Ziziphus jujuba Mill.) and expression in response to abiotic stress
Source: BMC Genomics. 2022 Jun 13;23:438. doi: 10.1186/s12864-022-08630-5 (PMC9195466; doi:10.1186/s12864-022-08630-5)
Supplement: Supplementary file 5 — Additional file 5: Table S5. ZjBAM family protein interactions at high confidence (0.900). [file 12864_2022_8630_MOESM5_ESM.docx]

| T**able S5 ZjBAM family protein interactions at high confidence (0.900)** | | | | |
| --- | --- | --- | --- | --- |
| ZjBAM family | interaction protein in jujube | orthologs gene in *A. thaliana* | *A. thaliana* ID name | combined score |
| Zj.jz015515046 (ZjBAM1) | Zj.jz040083023 | AT1G69830 | AMY3 | 0.962 |
|  | Zj.jz039989054 | AT4G09020 | ISA3 | 0.931 |
|  | Zj.jz018223043 | AT5G64860 | DPE1 | 0.943 |
|  | Zj.jz003639090 | AT2G40840 | DPE2 | 0.905 |
|  | Zj.jz017401047 | AT5G11720 | AT5G11720 | 0.949 |
| Zj.jz044849113 (ZjBAM2) | Zj.jz040083023 | AT1G69830 | AMY3 | 0.969 |
|  | Zj.jz006119172 | AT4G25000 | AMY1 | 0.901 |
|  | Zj.jz018223043 | AT5G64860 | DPE1 | 0.940 |
|  | Zj.jz017401047 | AT5G11720 | AT5G11720 | 0.940 |
| Zj.jz029235020 (ZjBAM3) | Zj.jz017401047 | AT5G11720 | AT5G11720 | 0.958 |
|  | Zj.jz039989054 | AT4G09020 | ISA3 | 0.97 |
|  | Zj.jz018223043 | AT5G64860 | DPE1 | 0.971 |
| Zj.jz029235021 (ZjBAM4) | Zj.jz018223043 | AT5G64860 | DPE1 | 0.951 |
|  | Zj.jz017401047 | AT5G11720 | AT5G11720 | 0.957 |
|  | Zj.jz039989054 | AT4G09020 | ISA3 | 0.96 |
| Zj.jz013313009 (ZjBAM7) | Zj.jz018223043 | AT5G64860 | DPE1 | 0.936 |
|  | Zj.jz017401047 | AT5G11720 | AT5G11720 | 0.956 |
| Zj.jz040841049 (ZjBAM8) | Zj.jz000817059 | AT2G39930 | ISA1 | 0.906 |
|  | Zj.jz039989054 | AT4G09020 | ISA3 | 0.974 |
|  | Zj.jz017401047 | AT5G11720 | AT5G11720 | 0.956 |
|  | Zj.jz018223043 | AT5G64860 | DPE1 | 0.978 |
