# Supplementary material for: Genome-wide identification of BAM (β-amylase) gene family in jujube (Ziziphus jujuba Mill.) and expression in response to abiotic stress
Source: BMC Genomics. 2022 Jun 13;23:438. doi: 10.1186/s12864-022-08630-5 (PMC9195466; doi:10.1186/s12864-022-08630-5)
Supplement: Supplementary file 6 — Additional file 6: Table S6. Annotation of ZjBAM family and corresponding orthologs in A. thaliana. [file 12864_2022_8630_MOESM6_ESM.docx]

| **Table S6 Annotation of ZjBAM family and corresponding orthologs in *A. thaliana*** | | |
| --- | --- | --- |
| ZjBAM family | orthologs gene in *A. thaliana* | Annotation in *A. thaliana* |
| Zj.jz015515046 (ZjBAM1) | AT4G17090  (CT-BMY) | https://smart.embl.de/smart/DDvec.cgi?smart=548:Pfam_Glyco_hydro_14(89\|506)+  Beta-amylase 3, chloroplastic; Beta-amylase activity. No alpha-amylase activity. Involved in cold resistance. Mediates the accumulation of maltose upon freezing stress, thus contributing to the protection of the photosynthetic electron transport chain. Plays a role in the circadian-regulated starch degradation and maltose metabolism in chloroplasts, especially at night. More active on phosphorylated glucan. Interacts directly with starch or other alpha-1,4-glucan; Belongs to the glycosyl hydrolase 14 family |
| Zj.jz044849113 (ZjBAM2) | AT3G23920  (TRBAMY) | <https://smart.embl.de/smart/DDvec.cgi?smart=575:Pfam_Glyco_hydro_14(109\|534)>+  Beta-amylase 1, chloroplastic; Beta-amylase activity. Can use p-nitrophenyl maltopentaoside (PNPG5) as substrate only in reduced form. Can play a minor role in the starch degradation and maltose metabolism in chloroplasts during the night. More active on phosphorylated glucan. Interacts directly with starch or other alpha-1,4-glucan |
| Zj.jz029235020 (ZjBAM3) | AT4G00490  (BMY9) | <https://smart.embl.de/smart/DDvec.cgi?smart=542:Pfam_Glyco_hydro_14(98\|518)>+  Beta-amylase 2, chloroplastic; Encodes a chloroplast beta-amylase. The enzyme activity is very weak compared to BAM1 and BAM3. Mutant of BAM2 has no visible phenotype |
| Zj.jz029235021 (ZjBAM4) | AT2G45880  (BAM7) | <https://smart.embl.de/smart/DDvec.cgi?smart=691:Pfam_BES1_N(66\|219)+Pfam_Glyco_hydro_14(251\|671)>+  Beta-amylase 7 (BAM7); Its function is described as cation binding, beta-amylase activity, catalytic activity; Involved in cellulose biosynthetic process, carbohydrate metabolic process, polysaccharide catabolic process; Located in cellular_component unknown; Expressed in 19 plant structures; Expressed during 12 growth stages; Contains the following InterPro domains: Glycoside hydrolase, family 14, conserved site (InterPro:IPR018238), Glycoside hydrolase, family 14 (InterPro:IPR001554), Glycoside hydrolase, catalytic core (InterPro:IPR017853), Glycoside hydrolase, subgroup, catalytic c [...] |
| Zj.jz013313009 (ZjBAM7) | AT2G32290  (BAM6) | <https://smart.embl.de/smart/DDvec.cgi?smart=577:Pfam_Glyco_hydro_14(81\|502)>+  Beta-amylase 6 (BAM6); Its function is described as cation binding, beta-amylase activity, catalytic activity; Involved in cellulose biosynthetic process, carbohydrate metabolic process, polysaccharide catabolic process; Located in chloroplast; Expressed in 14 plant structures; Expressed during 8 growth stages; Contains the following InterPro domains: Glycoside hydrolase, family 14, conserved site (InterPro:IPR018238), Glycoside hydrolase, family 14 (InterPro:IPR001554), Glycoside hydrolase, catalytic core (InterPro:IPR017853), Glycoside hydrolase, family 14B, plant (InterPro:IPR001371 [...] |
| Zj.jz040841049 (ZjBAM8) | AT5G45300  (BMY2) | <https://smart.embl.de/smart/DDvec.cgi?smart=689:Pfam_BES1_N(83\|232)+Pfam_Glyco_hydro_14(258\|669)>+  Beta-amylase 2 (BMY2); Its function is described as cation binding, beta-amylase activity, catalytic activity; Involved in cellulose biosynthetic process, carbohydrate metabolic process, polysaccharide catabolic process; Located in cellular_component unknown; Expressed in 22 plant structures; Expressed during 13 growth stages; Contains the following InterPro domains: Glycoside hydrolase, family 14 (InterPro:IPR001554), Glycoside hydrolase, catalytic core (InterPro:IPR017853), Glycoside hydrolase, subgroup, catalytic core (InterPro:IPR013781), BZR1, transcriptional repressor (InterPro:I [...] |
| Zj.jz004069034  (ZjBAM9) | AT5G18670  (BMY3) | <https://smart.embl.de/smart/DDvec.cgi?smart=536:Pfam_Glyco_hydro_14(90\|497)>+  Inactive beta-amylase 9; Putative beta-amylase BMY3 (BMY3) |
