# Supplementary material for: Genome-wide identification of BAM (β-amylase) gene family in jujube (Ziziphus jujuba Mill.) and expression in response to abiotic stress
Source: BMC Genomics. 2022 Jun 13;23:438. doi: 10.1186/s12864-022-08630-5 (PMC9195466; doi:10.1186/s12864-022-08630-5)
Supplement: Supplementary file 7 — Additional file 7: Table S7. List of primer sequences used in this study. [file 12864_2022_8630_MOESM7_ESM.docx]

|  | **Table S7 List of primer sequences used in this study** | | | |
| --- | --- | --- | --- | --- |
| Gene name | |  | Primer name | Primer sequence（5’-3’） |
| *ZjBAM1* | | qRT-PCR | F | AGGAGAAACCCAGAGTACATTTC |
|  |  |  | R | GTCACGGAAGCTCCTCATATAAT |
| *ZjBAM2* | |  | F | GCAGTCATGTCCTTCCATCA |
|  |  |  | R | CTCGGTCTCTGTCAATCTCTTC |
| *ZjBAM3* | |  | F | TCTGGCAATGGTGAGCTATG |
|  |  |  | R | GTGTATCTGCAACCTGGCTAT |
| *ZjBAM4* | |  | F | TCAGATGGTGCGAGAGACTAA |
|  |  |  | R | GATTGCGTCTTCCCTCTCTATC |
| *ZjBAM5* | |  | F | CGTCGAGCTAGCAAGAAAGT |
|  |  |  | R | GAGGAAGAGGAATCCAATGAGG |
| *ZjBAM6* | |  | F | CGTCGAGCTAGCAAGAAAGT |
|  |  |  | R | GAGGAAGAGGAATCCAATGAGG |
| *ZjBAM7* | |  | F | CAGAGTACCAACTGCCCATT |
|  |  |  | R | TGGGAGCATCACATAGATTGG |
| *ZjBAM8* | |  | F | CCTCCATCTCAACTGACAACA |
|  |  |  | R | ATGGTGGCTGACTGTCTAATG |
| *ZjBAM9* | |  | F | AGACAAGTCAGGACAGCATTAC |
|  |  |  | R | AAATACCCTGGATTGTCGAACC |
| *ACTIN9* | |  | F | CATGAAGGTTCCGAACCTAAGGAGC |
|  |  |  | R | CATGAAGGTTCCGAACCTAAGGAGC |
| *CYP* | |  | F | ACTGCACTATAAGGGCTCCACCTTC |
|  |  |  | R | GGAACCATTAGTGTTTGGTCCAGCA |
| *ZjBAM1* | | Target genes PCR amplification | F | gagaacacgggggactctagaATGGCTCTCACGTTACGTTC |
|  |  |  | R | ctccatcccgggagcggtaccCACAAGAGCAGCCTCCTTC |
| *ZjBAM7* | |  | F | gagaacacgggggactctagaATGATGTCCCCTGAAGAAAGTG |
|  |  |  | R | ctccatcccgggagcggtaccCTTAAATATTGAAAATAACTTGTTTACCAAATTAGC |
| *ZjBAM8* | |  | F | gagaacacgggggactctagaATGAACGACGACAGCCTG |
|  |  |  | R | ctccatcccgggagcggtaccGGATACAAGATCACCTGCTATTTCAC |
| *ZjAMY3* | |  | F | gAGAACACGGGGGACTCTAGAATGTCGACCGTTAACATAAAGC |
|  |  |  | R | GTACATCCCgggagcggtaccGGATAACTCCCAGACCTTATAGTC |
| *ZjDPE1* | |  | F | gAGAACACGGGGGACTCTAGAATGGCTATCGCAGGCTCATTC |
|  |  |  | R | GTACATCCCgggagcggtaccCAGTCGTCCATACGTTGACAAAATATC |
