# Supplementary material for: Genome-wide identification of BAM (β-amylase) gene family in jujube (Ziziphus jujuba Mill.) and expression in response to abiotic stress
Source: BMC Genomics. 2022 Jun 13;23:438. doi: 10.1186/s12864-022-08630-5 (PMC9195466; doi:10.1186/s12864-022-08630-5)
Supplement: Supplementary file 8 — Additional file 8: Table S8. The experimental design: a split-plot design for two-factor treatments. [file 12864_2022_8630_MOESM8_ESM.docx]

| **Table S8** **The experimental design: a split-plot design for two-factor treatments** | | |
| --- | --- | --- |
|  | Natural atmospheric temperature | Elevated temperature |
|  | T1 | T2  [T1 ＋ (2.0 ℃±0.5 ℃)] |
| Normal soil moisture (D1) | D1T1 | D1T2 |
| Moderate drought (D2) | D2T1 | D2T2 |
| Sever drought (D3) | D3T1 | D3T2 |
