# Supplementary material for: Genome-wide identification of BAM (β-amylase) gene family in jujube (Ziziphus jujuba Mill.) and expression in response to abiotic stress
Source: BMC Genomics. 2022 Jun 13;23:438. doi: 10.1186/s12864-022-08630-5 (PMC9195466; doi:10.1186/s12864-022-08630-5)

**Figure S1 Amplification of target genes, vector identification, and transformation of *Agrobacterium* strain GV3101 followed by PCR identification.** Target genes amplification **(a–e)**, vector *pCAMBIA1300YNE* PCR identification **(f–j)**, *Agrobacterium* strain GV3101 transform PCR identification **(k–o)** of *ZjBAM1*, *ZjAMY3*, *ZjBAM7*, *ZjBAM8*, and *ZjDPE1*, respectively. m: 0.7% agarose, 1 kb Marker; M: 1.5% agarose, DL2000 Marker; **(f–j)**: 1–8: single colony of *Escherichia coli* strain; **(k–o)**: 1–6: single colony of *Agrobacterium* strain, 7: ddH2O negative control, 8: plasmid *pCAMBIA1300YNE*, positive control.


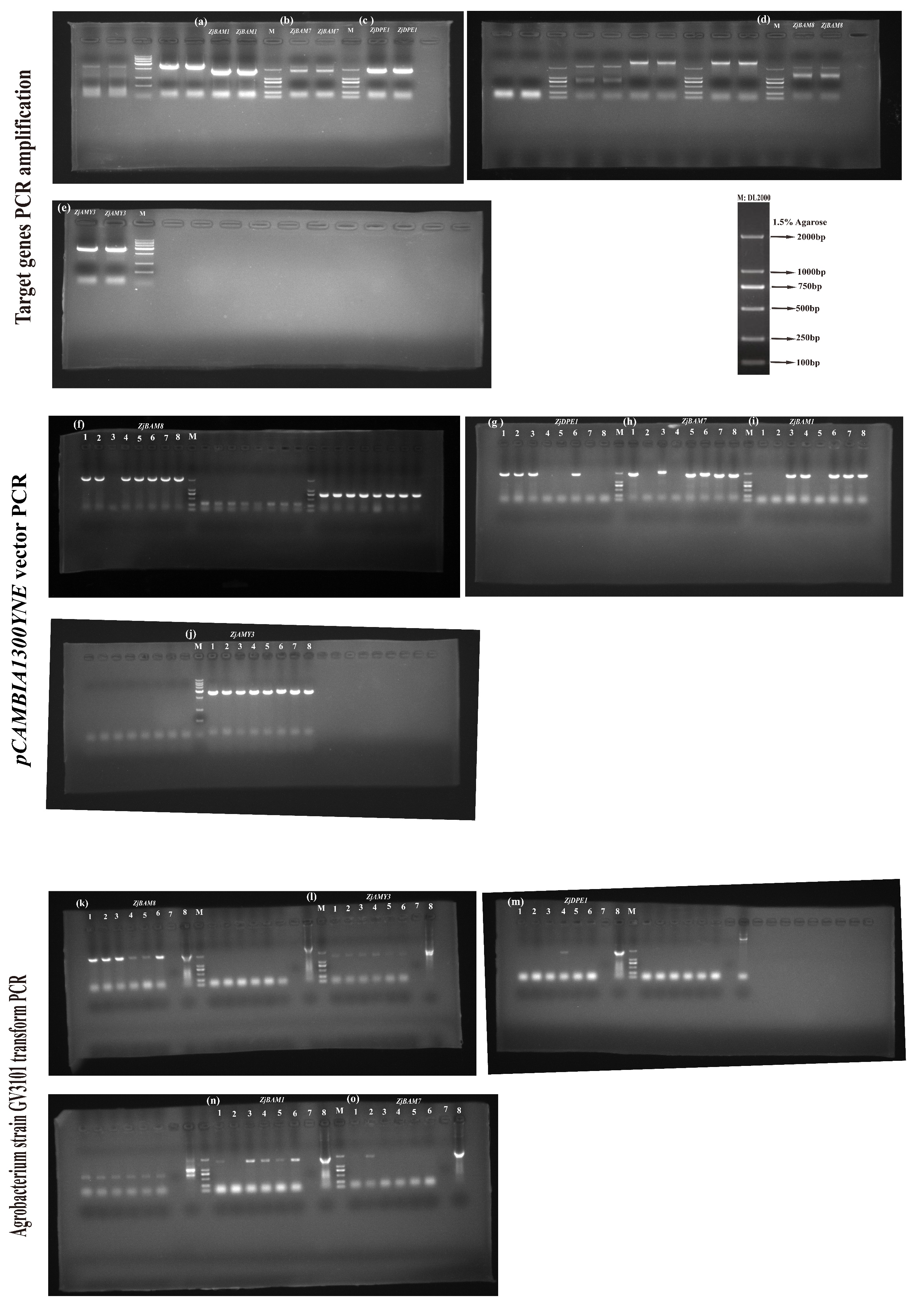

Supplement: Supplementary file 9 — Additional file 9: Figure S1. Amplification of target genes, vector identification, and transformation of Agrobacterium strain GV3101 followed by PCR identification. [file 12864_2022_8630_MOESM9_ESM.docx]
